# New evidence for an early settlement of the Yucatán Peninsula, Mexico: The Chan Hol 3 woman and her meaning for the Peopling of the Americas

**DOI:** 10.1371/journal.pone.0227984

**Published:** 2020-02-05

**Authors:** Wolfgang Stinnesbeck, Samuel R. Rennie, Jerónimo Avilés Olguín, Sarah R. Stinnesbeck, Silvia Gonzalez, Norbert Frank, Sophie Warken, Nils Schorndorf, Thomas Krengel, Adriana Velázquez Morlet, Arturo González González

**Affiliations:** 1 Institut für Geowissenschaften, Universität Heidelberg, Im Neuenheimer Feld, Heidelberg, Germany; 2 School of Biological and Environmental Sciences, Liverpool John Moores University, Liverpool, United Kingdom; 3 Department of Archaeology and Anthropology, Bournemouth University, Poole, United Kingdom; 4 Museo del Desierto, Carlos Abedrop Dávila, Nuevo Centro Metropolitano de Saltillo, Saltillo, Coahuila, Mexico; 5 Staatliches Museum für Naturkunde Karlsruhe, Geowissenschaftliche Abteilung, Erbprinzstrasse, Karlsuhe, Germany; 6 Institut für Umweltphysik, Universität Heidelberg, Im Neuenheimer Feld, Heidelberg, Germany; 7 Instituto Nacional de Antropología e Historia, CINAH Campeche, Campeche, Mexico; Max Planck Institute for the Science of Human History, GERMANY

## Abstract

Human presence on the Yucatán Peninsula reaches back to the Late Pleistocene. Osteological evidence comes from submerged caves and sinkholes (cenotes) near Tulum in the Mexican state of Quintana Roo. Here we report on a new skeleton discovered by us in the Chan Hol underwater cave, dating to a minimum age of 9.9±0.1 ky BP based on ^230^Th/U-dating of flowstone overlying and encrusting human phalanges. This is the third Paleoindian human skeleton with mesocephalic cranial characteristics documented by us in the cave, of which a male individual named Chan Hol 2 described recently is one of the oldest human skeletons found on the American continent. The new discovery emphasizes the importance of the Chan Hol cave and other systems in the Tulum area for understanding the early peopling of the Americas. The new individual, here named Chan Hol 3, is a woman of about 30 years of age with three cranial traumas. There is also evidence for a possible trepanomal bacterial disease that caused severe alteration of the posterior parietal and occipital bones of the cranium. This is the first time that the presence of such disease is reported in a Paleoindian skeleton in the Americas. All ten early skeletons found so far in the submerged caves from the Yucatán Peninsula have mesocephalic cranial morphology, different to the dolicocephalic morphology for Paleoindians from Central Mexico with equivalent dates. This supports the presence of two morphologically different Paleoindian populations for Mexico, coexisting in different geographical areas during the Late Pleistocene-Early Holocene.

## 1. Introduction

Osteological evidence for early American settlers is scarce and usually fragmentary, with only a few individuals known from both North and South America securely predating 10 thousand years (ky) ago ([1] and references therein). Mexico has long played a minor role in the discussion of the early settlement of the continent because researchers interested in the theme were majorly not aware of the wealth of Paleoindian skeletons found in this large geographical area of the Americas. Nevertheless, this situation is slowly changing [2]; today scientific interest particularly focuses on the Yucatán Peninsula (YP) in southern Mexico where a total of nine well-preserved human skeletons have been discovered during the past decade in submerged caves of the Tulum area and have been dated to between 13–9 ky BP ([1, 3–6]; S1 Table).

**Fig 1:**
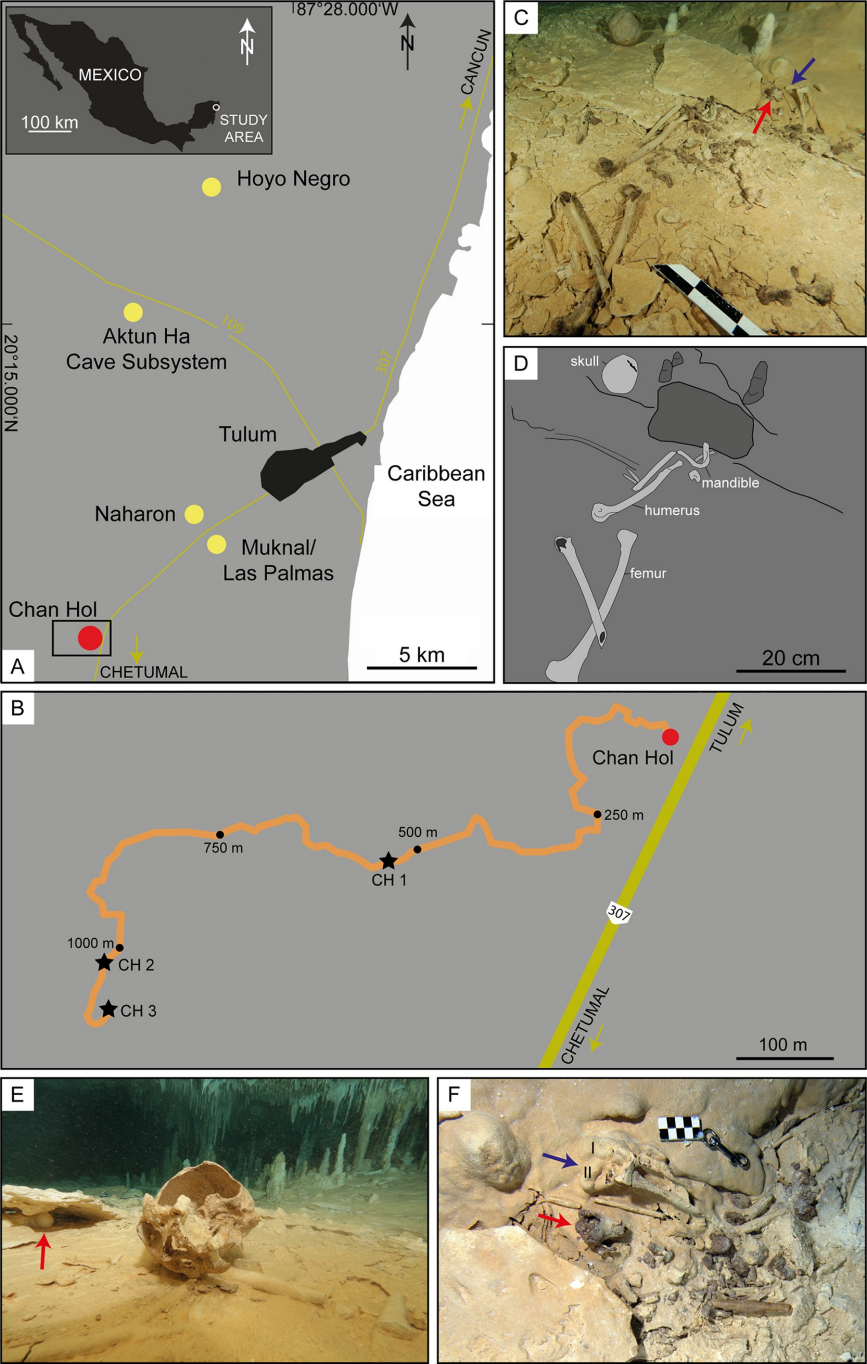
Geographical location of the Chan Hol 3 anthropological site. (A) Location of submerged caves containing human skeletal remains dating to >9 ky BP in the Tulum area of Quintana Roo, Mexico. Yellow dots refer to anthropological sites mentioned in the text with presence of human remains [1, 3–6]. The red dot marks the Chan Hol cave described previously by González González et al. [3, 6] and Stinnesbeck et al. [5]. (B) Close-up of the black box seen in Fig 1A with location of the three human skeletons found within the Chan Hol cave. (C) The Chan Hol 3 anthropological site. Note that human bones are spread over an area of 3 x 1 m. The original anatomical position of the skeleton is thus not preserved. The red arrow points to distal radius fragment while the blue arrow indicates position of finger bones (metacarpals, phalanges) depicted in Fig 2. A prominent flat limestone rock 0.3 wide and 0.2 m long and 50 mm in thickness is seen in the upper right quadrant of the photo, and the mandible immediately in front of this rock slab. (D) Interpretative drawing of the site. (E) The skull rotated upside down. It is likely this was water-transported and rolled for about 0.5 m to this position. The red arrow points to a broken stalagmite below the limestone slab seen in Fig 1C and 1D. (F) Flowstone encrusting phalangeal bones used for ^230^Th/U-dating of the Chan Hol 3 skeleton (see Fig 2 for details).

The caves are located within a few kilometers distance from the Caribbean coast and were dry and accessible during most of the period of interest of this study (13–9 ky BP), as they were not flooded until the worldwide sea-level rise that happened during the early Holocene (e.g. [4, 7–9]). The discovery of a well-preserved Paleoindian skull of a young girl from the submerged Hoyo Negro (Black Hole) sinkhole (Fig 1A) has received special interest. The individual was ^14^C-dated to 10,976±20 y BP (12,910–11,750 cal y BP; 95.4% probability using CalPal) by Chatters et al. [1] based on bioapatite from tooth enamel. Previously, a similar ^14^C age was already published for a human skeleton from Naharon cave (Fig 1A), also located close to Tulum, with an age of 11,570±65 ^14^C y BP (13,571–13,337 cal y BP; 68% probability using CalPal) [3, 6]. It is, however, difficult to exactly determine the ^14^C age of these two humans using conventional radiocarbon dating, because the amount of collagen found in their bones and teeth is extremely low. This is due to a general lack of collagen preservation in human and faunal remains found underwater in the Tulum caves (e.g. [4, 5]), which has been interpreted as the result of exposure of the osteological remains for thousands of years to alternating salt- and fresh water environments [5]. In addition, bioapatite is highly susceptible to contamination with fossil carbon resulting in false, mostly older ages [1]. Therefore, Stinnesbeck et al. [5] dated a stalagmite that had precipitated on top of a human pelvis earlier discovered in the Chan Hol cave system [3]. The analysis of uranium-thorium isotopes of the stalagmite precipitated on the Chan Hol 2 skeleton resulted in a minimum age of 11.3 ky BP for this human skeleton. However, the correlation of oxygen and carbon isotope ratios in the speleothem with other regional, independently dated paleoclimate records, indicates a much earlier onset of speleothem growth, suggesting an older age of about 13 ky BP for the Chan Hol 2 individual [5]. This implies that Chan Hol 2 is one of the oldest known skeletons of the American continent [5].

**Fig 2:**
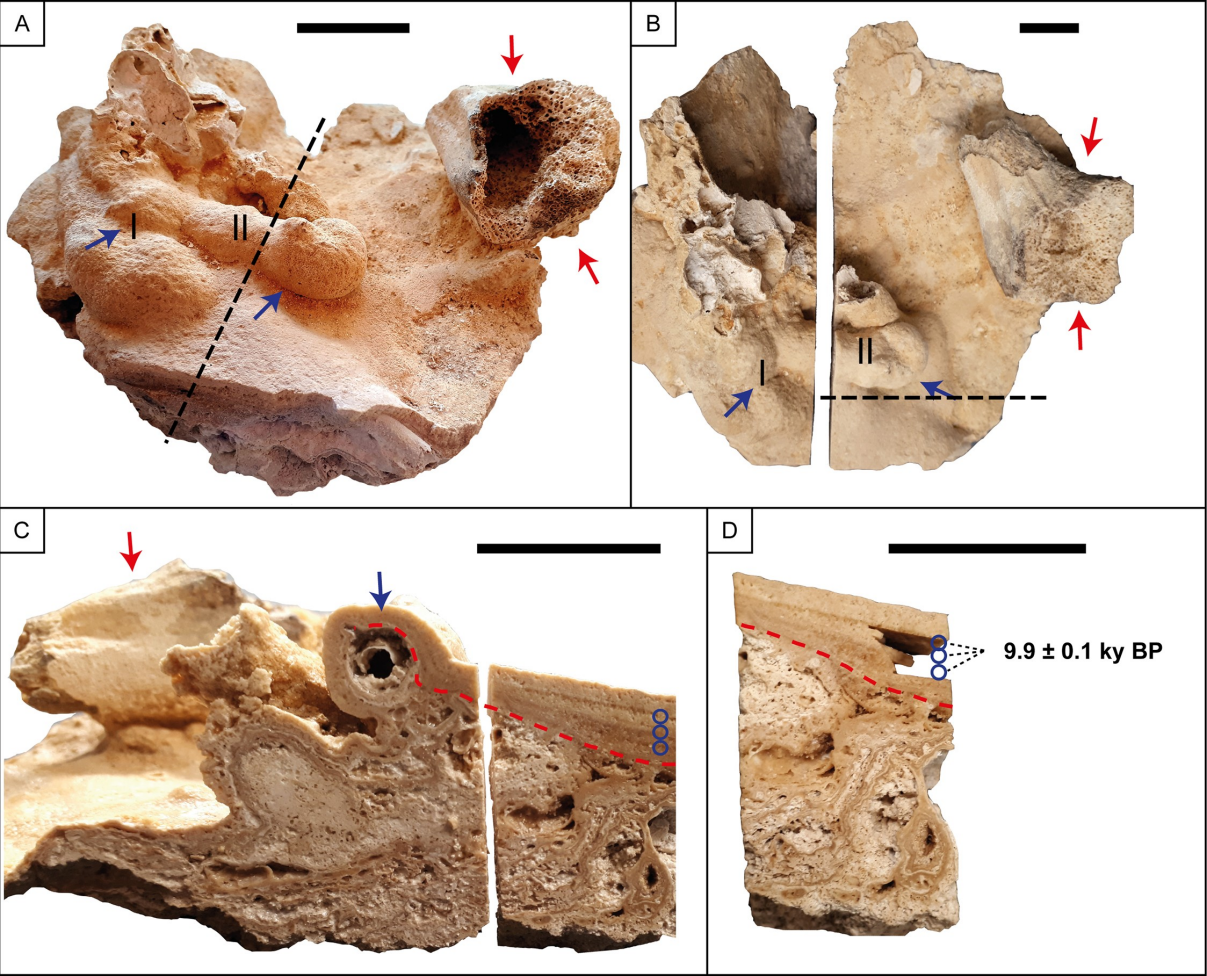
Flowstone encrusting phalangeal bones of the Chan Hol 3 human individual. For the original position of the sample on the cave floor see Fig 1C and 1F. Red arrows point to the position of distal radius and blue arrows to carpal and metacarpal bones, and blue circles to the position of samples used for ^230^Th/U-dating. (A) Sample seen from above. The dotted line indicates the position in which the sample was vertically cut. Note lateral view of distal radius fragment. The covered bone to the left is a carpal (I) and the one next to it is a metacarpal (II). (B) view of distal radius shown in (A) from above. (C) Vertical cross section of the sample. Note that the blue arrow points to the spherical aperture which originally represented the metacarpal and is now dissolved. (D) Magnification of the slab shown on the right side of (C), with the position of the three ^230^Th/U-dating samples with approximately coeval ages of 9.9±0.1 ky. A contaminating ^230^Th/^232^Th activity ratio of 3.96±0.09 is estimated from the Osmond isochron. Scale used in all figures is 20 mm.

We here report on a new skeleton from the Chan Hol cave, named Chan Hol 3, which is the third human skeleton discovered from the Chan Hol system [3, 5]. The cave also preserves evidence for early to mid-Holocene human usage in the form of numerous charcoal accumulations with radiocarbon dates between 8110 ± 28 ^14^C y BP (9122–8999 cal y BP) to 7177 ± 27 ^14^C y BP (8027–7951 cal y BP) [9].

## 2. Geological setting

The Tulum submerged cave system on the northeastern YP in the Mexican state of Quintana Roo, is among the most extensive active underwater cave systems worldwide, with a presumed total length of 7,000 km, but only 1,500 km have currently been explored [10]. Karst developed in almost horizontally layered, thick-bedded, shallow-water carbonate bedrock of Neogene ages and is the result of intensive development during the Pleistocene, caused by a series of sea-level oscillations and changes in the overall hydrology of the area [11, 12]. Sea-level rise on the YP was predominantly controlled by eustasy, as the peninsula has been tectonically stable in the recent past and glacial isostatic adjustments are negligible in this tropical area [12–14].

During the Last Glacial Maximum (25 to 19 ky BP) sea-level was more than 100 m below the present day sea-level and large parts of the Tulum cave system were dry and accessible for animals and humans. During the last deglaciation, between 13 and 7.6 ky BP, sea-level rose again and modern water levels were reached at approximately 4.5 ky BP [7–9], although oscillations of up to a few meters are known to have occurred during Mayan times, and later [15]. Today, the Tulum cave system contains a coastal, density stratified aquifer, i.e. a freshwater layer overlying penetrating seawater. The depth of the halocline depends on the global sea-level as well as on the thickness of the superimposed freshwater layer. It is controlled by the distance to the coastline as well as the amount of precipitation, with a hydraulic gradient across the YP of between 0.5 and 100 mm/km. As most of the Tulum caves are hydrologically open-systems, groundwater flows through the porous limestone karst directly towards the ocean. In consequence, water level near the coast (e.g. in the Tulum area) is approximately equivalent to mean sea-level ([15] and references therein).

### 2.1. The Chan Hol 3 Site and the Human Skeleton

The Chan Hol 3 skeleton was found in September 2016 by cave explorers Vicente Fito and Ivan Hernández during a systematic survey led by Jeronimo Avilés in Chan Hol cave. The entrance to the cave is at Chan Hol cenote (sinkhole), located at 20°9.467’ N, 87°34.165 W, about 15 km southwest of Tulum, and about 11.5 km from the coastline (Fig 1). The skeleton was discovered in a low cave tunnel in fresh water at 8 m water depth, at 1141 m diving distance from the cenote (Fig 1). During the dive, the sites of the Chan Hol 1 [3] and Chan Hol 2 [5] human skeletons were passed at 541 m (Chan Hol 1) and 1027 m (Chan Hol 2) from the cenote entrance. The maximum depth of the Chan Hol cave is about 13 m below present day sea-level and the halocline is located at about 9 m water depth. Due to its shallow position, this part of the Chan Hol cave must have been accessible until early stages of the middle Holocene [5, 9]. Anthropogenic charcoal accumulations in the cave have been dated to between 8,110±28 ^14^C y BP (9,122–8,999 cal y BP) and 7,177±27 ^14^C y BP (8,027–7,951 cal y BP) [9].

## 3. Material and methods

Underwater registration and documentation of skeletal remains in the Tulum underwater caves has been described in detail [6]. The underwater documentation has been executed by J.A.O., Vicente Fito, Eugenio Acévez and Ivan Hernández. After collection, the skeleton was treated with distilled water for eight months and slowly dried. Underwater photographs were taken with an Eos rebel Ti4 with a 10–20 mm zoom lens inside an ikelite housing. Laboratory photography was taken with a Canon D5 Mark III with 50 mm and 100 mm macro lenses.

### 3.1. Human osteology

The cranium is well preserved allowing for the execution of detailed craniometric measurements of Howells [16] and Buikstra and Ubelaker [17]. Cranial and femoral measurements are listed in S2 Table. Paleopathological analysis and documentation follow definitions made by Ortner and Putschar [18] and Aufderheide et al. [19]. Sex, age, and stature assessments were completed following procedures established by Buikstra and Ubelaker [17], Genovés [20], and Walker [21]. Stature equation was chosen because the parent sample was from Mexico [20].

Cranial Indices and Upper Facial Indices were calculated to make overall comparisons between crania across North, Central, and South America. We excluded the Hoyo Negro skull as there are no data published currently for this individual. For other individuals, a full Principal Components Analysis (PCA) was calculated using samples from the Howell’s cranial database [16, 21–23] and other open source cranial data [24–28]. A total of 452 human skulls from ten different samples were included for analysis (S3 Table). Twelve variables were used and were all collected from peer-reviewed articles and books [16, 21, 22, 24–28]. The 12 variables were chosen because they were most frequently available from all specimens. For individuals that were missing these variables, a *k* Nearest Neighbor analysis was used to compute what the missing variables would be [29]. Each sample’s missing values were computed separately to allow for a better portrayal of that sample variation. S3 Table lists the variables used for analysis. From these 12 cranial variables (S3 Table), PCA was computed (S4 Table).

### 3.2. ^230^Th/U-dating

Samples for mass spectrometer ^230^Th/U-dating were taken from a flowstone up to 10 mm thick overlying and encrusting phalanges of the Chan Hol 3 skeleton (Fig 2). As the carbonate encrustation of the bone and cave floor itself has a very heterogeneous structure, our sampling strategy concentrated on this laminar layered flowstone from which a sequence of three subsequent samples was cut using a diamond wired band saw (Fig 2). This strategy ensured that samples were taken to minimize mixing of material of possibly different ages. Individual sample thickness is typically 2 mm (in growth direction), with an individual sample weight ranging between 60 and 90 mg. All samples were pre-cleaned through a weak acid leach and dried prior to dissolution in 7 N HNO_3_. The chemical preparation for mass spectrometric U and Th isotope measurements was conducted at the Institute of Environmental Physics at Heidelberg University using wet-column chemistry (resin: UTEVA^®^) to purify U and Th from the flowstone samples. The chemical protocol follows the one of Wefing et al. [30]. The natural isotopes of uranium (^238^U-^235^U-^234^U) and thorium (^232^Th- and ^230^Th) and the artificial isotopes of the triple-spike (^233^U-^236^U-^229^Th) were quasi-simultaneously analyzed using a multi-collector, inductively coupled, plasma source mass spectrometer (MC-ICP-MS) (Thermo Finnigan Neptune^Plus^) coupled to a desolvator (CETAC—ARIDUS) at the Institute of Environmental Physics, Heidelberg University [30, 31]. The 3 sample measurements of the Chan Hol 3 flowstone samples were bracketed with measurements of HU-1 reference material and prior to each standard a blank sample was analyzed. The detector yield and abundance sensitivity were independently assessed before and after the sample analysis and the data was corrected for instrumental biases, abundance sensitivity, detector yield and peak tailing. The raw data treatment is conducted using an in-house Matlab Script [31]. Ages were calculated using the half-lives of both elements [32]. Using the more recent values of Cheng et al. [33] does not influence the results.

Due to the complete dissolution of organic matter, specifically collagen, we refrained from the extraction of collagen and thus ^14^C age determination of the Chan Hol 3 individual.

### 3.3. Strontium Isotope analysis

Due to availability and preservation we selected the third left mandibular molar from Chan Hol 3 for Sr-isotope analysis. Third molar enamel forms during adolescence, between 7 and 16 years of age (e.g. [34]).

We sampled 2 to 5 mg chips of tooth enamel using a 0.2 mm diamond-coated cutting disc to minimize material loss of the valuable sample. We carefully avoided dentine components in the samples, because dentine is known to be sensitive to diagenesis and, unlike enamel, easily takes up mobile geogenic strontium components from the surrounding rocks and sediments [35].

The enamel pieces were cleaned by repeated washing with ultrapure water. After drying the enamel grains were digested using nitric acid. Strontium was purified through wet-column extraction chemistry using an EiChrom SrResin^®^ column [36]. The protocol applied at the Institute of Earth Sciences, Heidelberg University was adopted after Kober et al. [37]. A 1 ml column was filled with SrResin (TRISKEM) and washed with 6 column volumes (CV) H_2_O. Next the columns were loaded with 3 ml 7N HNO_3_. The samples were dried and re-dissolved in 1 ml nitric acid added onto the columns. The columns were then washed with 6 CV 7 N HNO_3_. To elute Sr from the resin the columns were rinsed with 3 CV H_2_O. The samples were evaporated on a hot plate until a small barely visible drop remained at the bottom of the beaker. The column chemistry was repeated to further purify Sr from the sample matrix and the final Sr solution was evaporated to dryness. The sample was re-dissolved in a drop of concentrated HNO_3_ and a drop of H_2_O_2_ to ensure dissolution of remains of the resin and it was then again evaporated to dryness. Finally, samples were dissolved in 10 μl 7N HNO_3_ and were transferred onto a preheated rhenium filament. Isotopic measurements (10 sequences of each 10 measurements) were conducted on a thermal ionization mass spectrometer (Finnigan MAT-262) using a dynamic multi-collection method normalized to the Nier value of ^86^Sr/^88^Sr = 0.1194 using an exponential fractionation law. All isotopes were measured on Faraday cups with minimum ^86^Sr intensities of 0.5 V. Each measurement was checked for ^85^Rb. Isotope ratios were corrected for internal mass fractionation assuming a stable ^88^Sr/^86^Sr ratio of 8.375209. The NIST isotope standard SRM-987 was used for routine monitoring and correction of instrumental bias and to assess reproducibility. Replicate SRM-987 analysis of the ^87^Sr/^86^Sr ratio yields 0.710261 ± 0.000006 (2σ, N = 4).

## 4. Results

### 4.1. The Chan Hol 3 site and skeleton

At the Chan Hol 3 anthropological site, human bones are spread over an area of 3 x 1 m and the original anatomical position of the skeleton is thus not preserved (Fig 1C and 1D). The skull is seen at about 0.5 m north of the rest of the skeleton (Fig 1C–1E), while forearm (radius, ulna) and finger bones (metacarpals, phalanges) are identified to one side of a rectangular shaped limestone slab 0.3 x 0.2 m wide and 50 mm in thickness, while a humerus and metacarpals are identified on the opposite side, along with the mandible. Both legs are fully extended and located in the south quadrant (Fig 1C and 1D), but the original position of these bones is disturbed.

**Fig 3:**
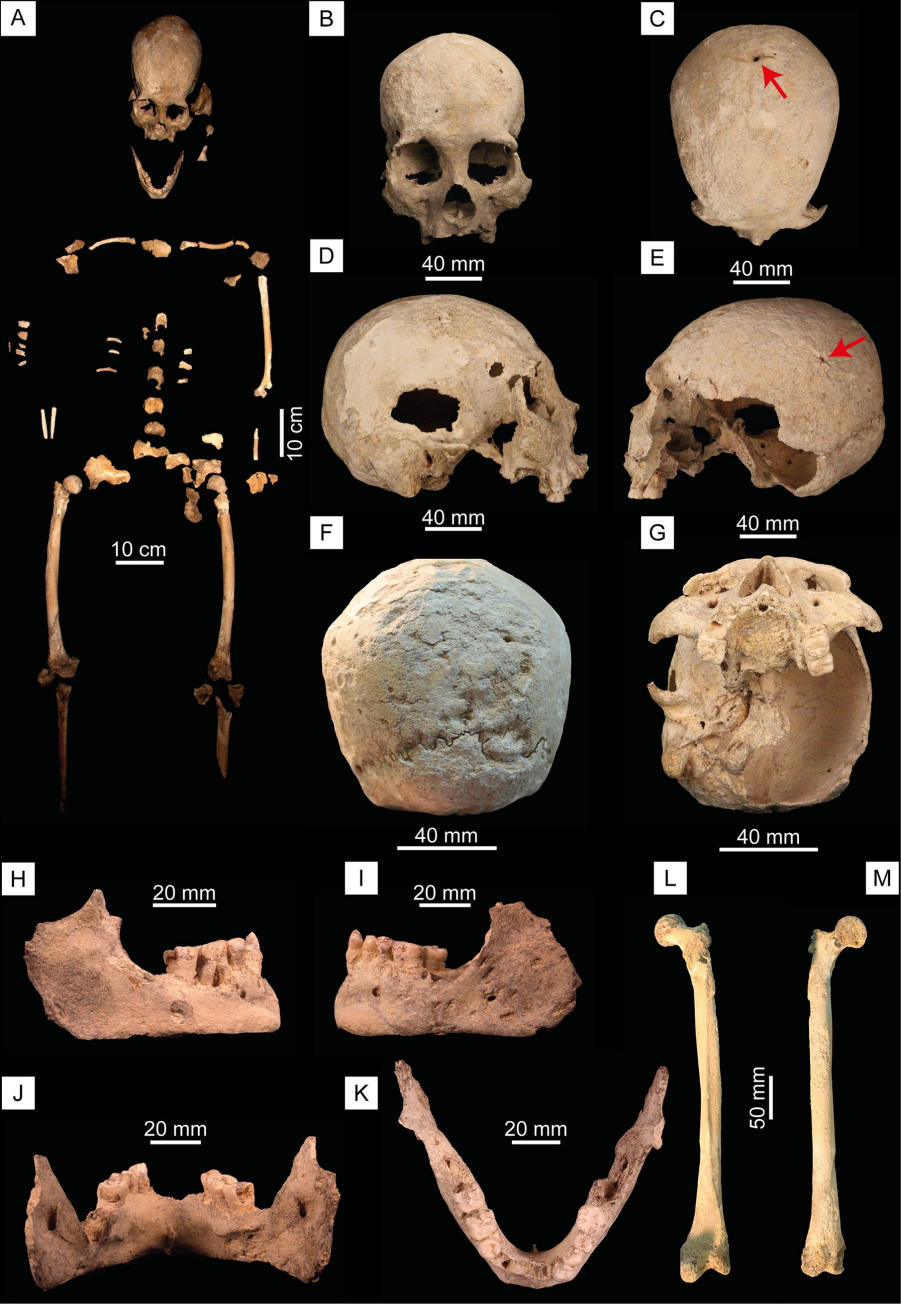
The Chan Hol 3 skeleton. (A) Bone map of the Chan Hol 3 skeleton. (B) Cranium in rostral view. (C) Cranium in dorsal view. Arrow points to a trauma on the posterior portion of the parietal. (D) Cranium in right lateral view. (E) Cranium in left lateral view. The red arrow points to trauma on the parietal bone. (F) Cranium in caudal view. Note extensive bone destruction on the occipital bone here interpreted to result from treponemal bacterial disease. (G) Cranium in ventral view. (H) Mandible in right lateral view. (I) Mandible in left lateral view. (J) Mandible in caudal view. (K) Mandible in dorsal view. (L) Right femur in frontal and (M) posterior view.

The Chan Hol 3 individual is only about 30% complete which indicates that many bones may either have been water-transported and carried away, were lost due to decomposition, or are still in the cave covered by flowstone. Bones collected include the cranium, mandible, both clavicles, manubrium, the left humerus, both femora, both tibiae, three fragments of the pelvis (ilium and ischium), two ulnae and one radial shaft fragment, seven fragments of vertebrae (thoracic and lumbar), seven fragments of ribs, and three phalanges (Fig 3).

The cranium is largely preserved but post-mortem fractures have occurred. Most internal cranial bones (ethmoid, vomer, and large amounts of the sphenoid etc.) are lost. Portions of the occipital bone, especially near the *foramen magnum*, are also missing. A small wormian bone was located alongside the right lambdoidal suture. The left parietal has been fractured post-mortem but remains present. The fracture margins remain sharp, suggesting that the fracture was recent. Zygomatic arches on both sides are no longer present.

The only teeth that are still present in the maxilla are the left and right 1^st^ molars and the left 2^nd^ molar (Fig 3G). There is post-mortem breakage on the right canine with the root still present in the maxilla. The four incisors have complete resorption indicating that they were lost ante-mortem alongside the right upper canine, left, and right premolars. The 2^nd^ and 3^rd^ molars on the upper right side have been lost, most likely ante-mortem due to dental abscess (Fig 3G).

The mandible is mostly complete, apart from the mandibular condyles being broken post-mortem (Fig 3H–3K). Central and medial incisors are no longer present showing ante-mortem loss with complete resorption of the alveolar bone alongside the loss of the right lower canine and 1^st^ premolar. The 2^nd^ and 3^rd^ molars from the left side and the 3^rd^ molar on the right are not present.

Postcranially, both clavicles are present alongside the manubrium which presents a recent post-mortem break running superior-inferiorly. The left humerus is presented as two main fragments, the humeral head and the diaphyses, and the distal portion (trochlea and capitulum). Several pieces of vertebral bodies and arches belong to thoracic and lumbar regions. The left *os coxa* has fragments from the ilium and ischium, with the right side only preserves the ilium. Both femora are present with only the right side preserving the femoral head (Fig 3L and 3M). The tibial diaphysis has been preserved for both left and right sides but shows more fragmentation on the left.

### 4.2. Biological profile

The Chan Hol 3 individual represents an adult female. Sex determination followed morphognostic traits of the skull [38] and of the greater sciatic notch [39]. Using Walker’s method for the assessment of the Greater Sciatic Notch [39], a score of 2 was given which is more indicative of a female individual. For sex diagnosis using the skull, Walker’s methodology [38] was used alongside that of Buikstra and Ubelaker [17]. The Glabella was scored to 2, Mastoid Process scored 2, Nuchal Crest scored 1, and the Mental Eminence scored 4. Sadly, the Supra-Orbital Margins could not be scored with confidence due to insufficient preservation. The femoral head diameter was taken and compared to the sectioning points laid out by Iscan and Steyn [40]; page 174. Musculoskeletal markings on the long bones are all weakly developed and overall present as gracile.

Chan Hol 3 is a fully mature adult showing complete fusion of all long bones and the medial clavicle suggesting an age of 21+ years. Due to fragmentation, the pubic symphysis and the auricular surface of the pelvis were not present for further age assessment following cranial suture closure. Due to slight erosion of the external surface of the skull, ectocranial suture closure techniques could not be fully implemented [41]. From what was available, the mid-lambdoidal and lambda sutures show signs of non-closure whilst the rest shows either significant closure or complete obliteration of the suture. All permanent dentition had erupted resulting in an age of >18 years. With the addition of using Brothwell’s method for aging using dental attrition [42], slight wear is identified on the occlusal surfaces of the 1^st^ and 2^nd^ mandibular molars. This puts Chan Hol 3 at the later phase of the first stage which provides an approximate age of 25 years. With this information, the Chan Hol 3 woman was a young adult (30±11 years old) at the time of death, but this assessment is to be taken with caution. Stature was calculated using the regression equation by Genovés using maximum femoral length [20]. With this, a stature of 1.635±0.035 m was calculated. Overall, Chan Hol 3 is therefore an adult female, approximately 30±11 years of age, with a height of 1.64 m.

### 4.3. Craniomorphology

**Fig 4:**
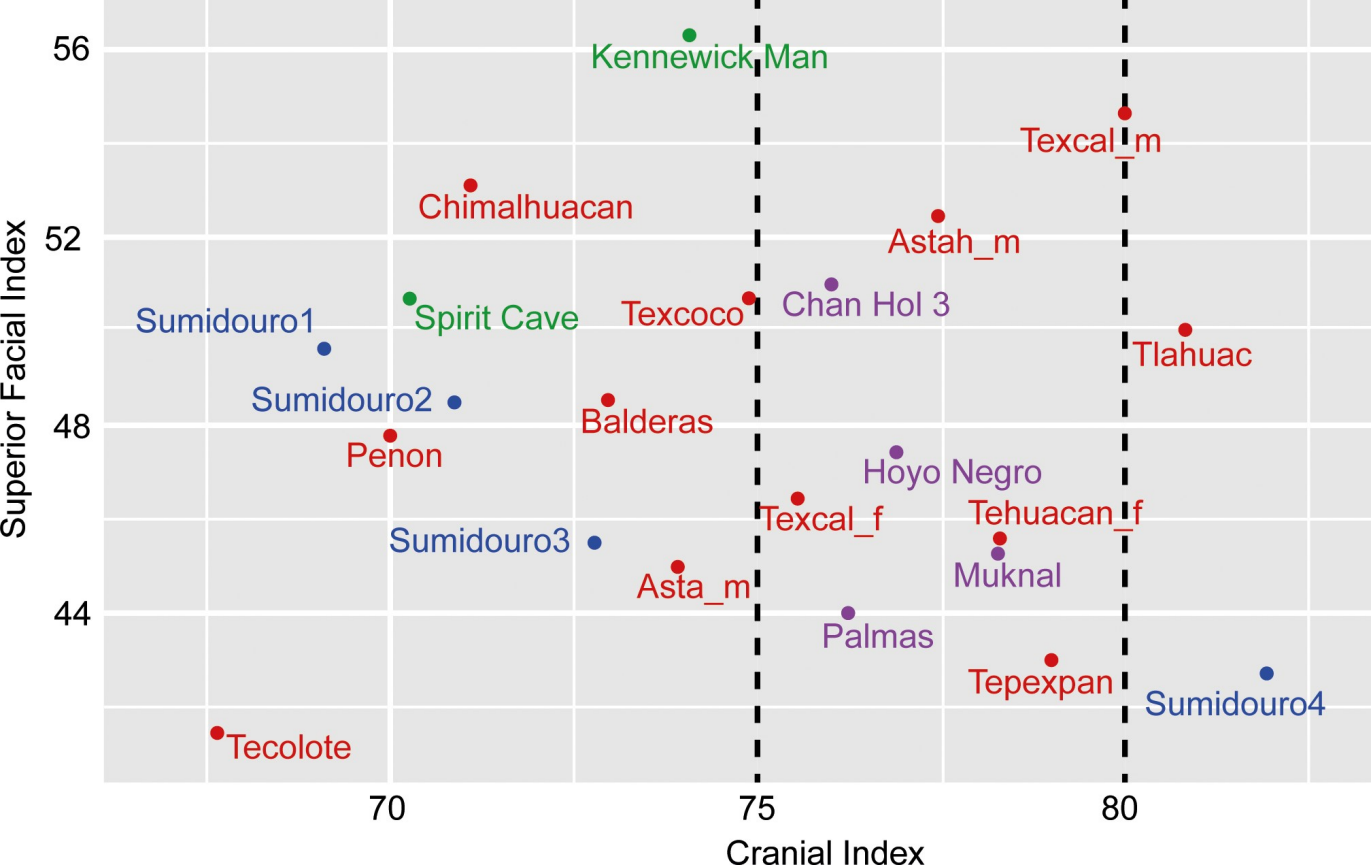
Cranial index of the Chan Hol 3 woman in comparison with other North and South American skeletons ranging in age to >9 ky BP. For numerical values of cranial index and for Superior Facies Index see S5 Table. Purple color: skeletons from the YP. Red color: skeletons from Central Mexico. Green color: skeletons from North America. Blue color: Skeletons from Lagoa Santa (Sumidouro Cave), Brazil. The data indicate that the Paleoindians from the YP are all in the mesocephalic index range (75–80), contrasting with the individuals from Central Mexico and North America with dates older than 9 ky BP which are in general dolicocephalic (68–75). Graph modified from Hernández Flores [43].

Cranial Index was calculated to 76.00 and resulted in Chan Hol 3 being classified as mesocephalic. This result fits in with the other three individuals from Yucatan: Hoyo Negro, Muknal and Las Palmas (Fig 4; S5 Table). However, this cannot be said for the large proportion of other crania in a similar age range (Late Pleistocene-Early Holocene), most of them being dolicocephalic (Fig 4; S5 Table). Chan Hol 3 has an upper/superior facial index of 51 which places the skull as having a medium face, neither broad nor narrow. This is slightly different to the other crania from Yucatan which have slightly broader faces (Fig 4; S5 Table). In general terms, however, our data indicate that two distinct morphologies were present in Mexico as early as 12 ky BP, with the individuals from Yucatan being the only sample present with mesocephalic skulls.

**Fig 5:**
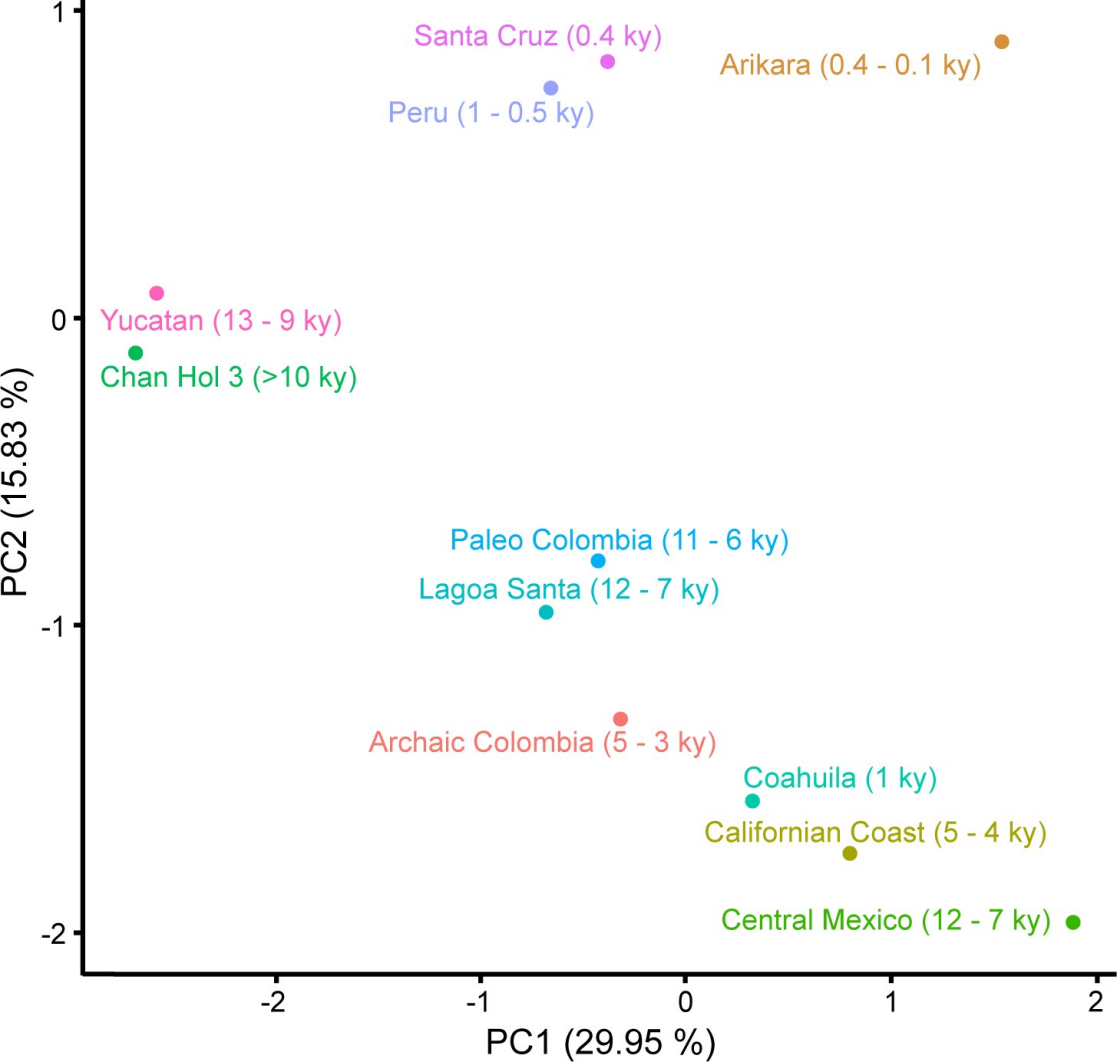
Principal Components Analysis based on crania from the Americas with PC1 explaining 29.95% and PC2 explaining 15.83% of the total variation seen within the samples. See S4 Table for PC1 and PC2 loadings for each variable.

For the PCA study, a total of 452 individuals were analyzed when pooling both males and females (Males: 241 & Females: 211; S3 Table). This data set included all specimens currently present from the YP, with the newly discovered Chan Hol 3 plotted separately to highlight its association with the rest of the Yucatan individuals. Two Principal Components (PCs) were extracted resulting in 29.95% of the variation being explained by PC1 and 15.83% being explained by PC2 (Fig 5). The main differences are seen along the second principal component where the three ‘younger’ samples (Arikara, Peru, and Santa Cruz) occupy space within the positive Y-axis, whereas older samples all have negative PC2 scores (Fig 5). This difference is primarily explained by the variation seen in five cranial measurements (S4 Table). The larger Maximum Cranial Length (GOL) and Basion—Bregma Height (BBH) are more associated with the older samples (Mexico and South America), whilst Maximum Cranial and Facial Breadths (XCB and XFB), and Nasio–Occipital Length (NOL) are greater in the younger samples. Focusing on the negative PC2 space, we see three main clusters among the South American, Mexican, and Californian samples. This variation is primarily driven by three measurements on the mid-facial skull. The three individuals from the YP differ from the other two clusters by smaller Bizygomatic Breadths (ZYB), Nasion-Prosthion Heights (NPH) and Nasal Heights (NHL) (Fig 5; S4 Table). We further identify a cluster containing the other two Mexican samples and the one from California; the three samples exhibit much larger ZYB, NPH, and NHL, alongside the South Americans, as compared to the sister Yucatan sample (Fig 5). These differences highlight some of the main differences found between a dolicocephalic skull from Central Mexico and a mesocephalic skull from Yucatan.

### 4.4. Pathologies

**Fig 6:**
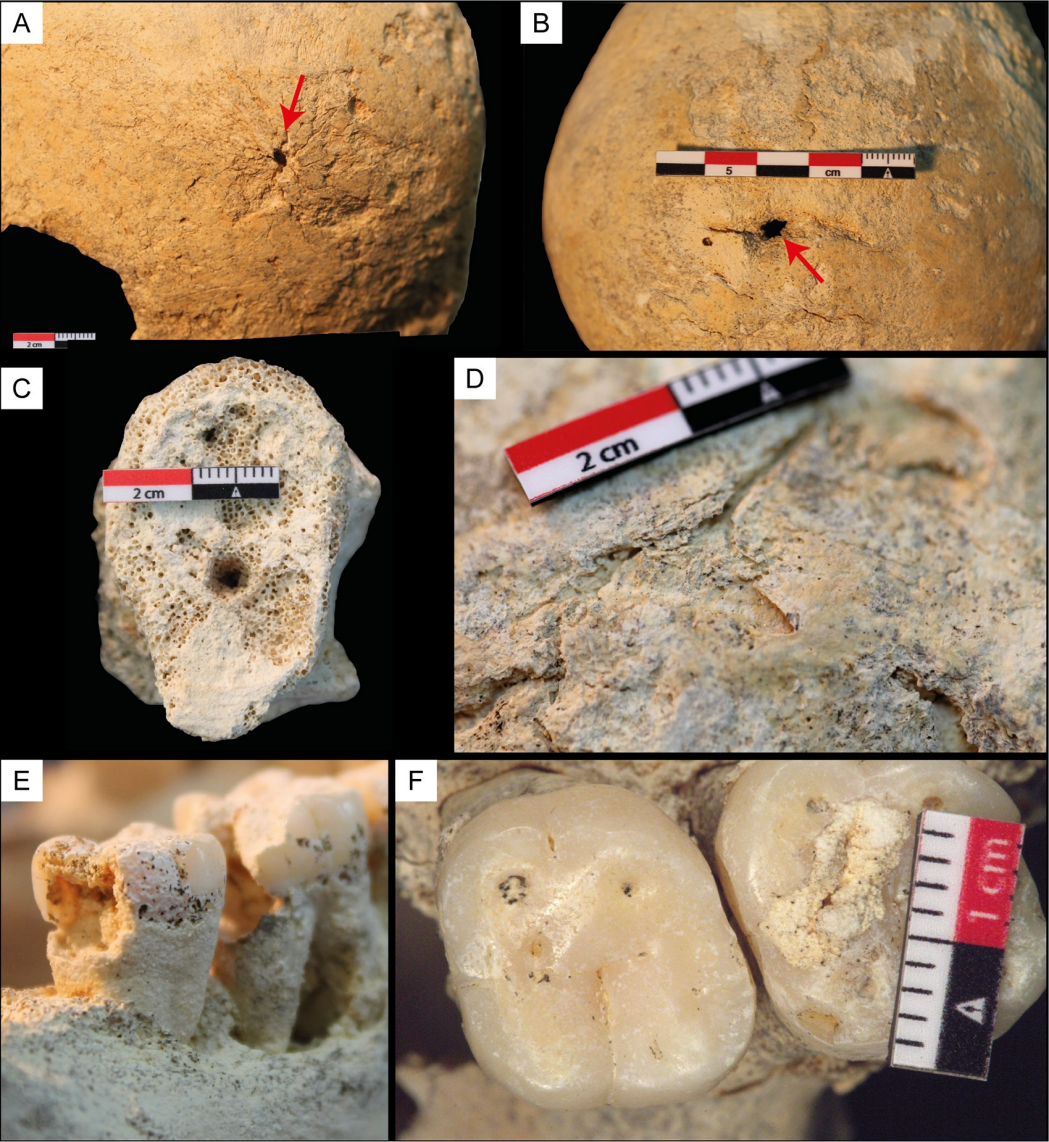
Pathologies detected in the Chan Hol 3 female. **(**A) The red arrow points to trauma on the left parietal bone. (B) The red arrow points to trauma on the parietal bone in caudal view. (C) Eburnation on the vertebrae. (D) Cut mark on the right temporal bone. (E) Caries in molars 2 and 3 in the right mandible in buccal view. (F) Plaque and caries in molars of the left maxilla in occlusal view.

Caries were identified on the first and second molars on the right side of the mandibular ramus (Fig 6E). Also, the interproximal spaces between the molars are affected. The third mandibular molar on the right has been completely resorbed. The third premolar on the right and left sides and the second molars present plaque. The mandibular ramus on the left side presents a heavy abscess and a loss of the third molar (Fig 3I). The abscess goes deep into the bone, which must have caused severe pain. An abscess is seen on the middle and right part of the mandible leading to a loss of all incisors and canines as well as the first premolar (Fig 3J and 3K).

The Chan Hol 3 presents arthritis on the humeral head and some vertebrae are showing signs of eburnation (Fig 6C). Schmorl’s nodes are present on two of the vertebral bodies and can be described as vertical disk hernias due to their position on the vertebral bodies.

The posterior portion of the cranial vault surface exhibits dents and crater-like deformations. These deformations appear to be pathological in nature, especially surrounding the lambdoidal suture (Fig 3F). The affected bone tissue has signs of a potential infection which may have been caused by the healing trauma to the cranial vault (Fig 3F). The facial skeleton is not affected. Possible differential diagnoses of the pathological lesions would be *Treponema peritonitis* alongside osteitis, or severe periostitis resulting from a trauma to the skull [18, 19, 44]. The lesions observed in Chan Hol 3 show similarities to a pre-Colombian South American male skull exhibiting *Treponema peritonitis* from 1650 y BP [22]. Other treponemal diseases such as Syphilis and Yaws are less likely because they typically manifest in the skeleton differently (facial skeleton not affected etc.) and there is a lack of *caries sicca*.

The Chan Hol 3 individual shows signs of three traumas to the posterior and lateral portion of the skull resulting in bone loss and remodeling. Two traumas are visible on the posterior portion of the right parietal bone along the sagittal and lambdoidal sutures and are caused by a potential sharp object (Figs 3C, 3F, 6A and 6B). The injuries resemble an isolated incisive trepanation which produces a fusiform groove (a.k.a. Indian canoe), which is normally caused by scraping [45]. A differential assessment would be a healed sharp force trauma that has signs of healing, with the perforations and slight irregular borders being caused by post-mortem taphonomic processes. Neither of the possible two sharp force traumas show signs of radiating/concentric fractures at the macro level due to the sharpness of the object hitting the skull and there creating an incised wound. As mentioned before, the slight irregular border/margins could have been caused by healing and post-mortem taphonomic processes. The third potential trauma is identified on the left parietal bone. This shows a circular perforation surrounded by a raised area of new bone which could be signs of healing (Figs 3E and 6A). The cause of this third trauma is unknown due to the new bone formation. Two small cut marks (20 mm) have also been identified on the right caudal side of the temporal lobe (Fig 6D). To help fully understand the pathologies and trauma seen on the cranium further analysis using medical imaging (computerized tomography/CT) would help with the diagnoses of such lesions.

### 4.5. Dating

The preservation of the Chan Hol 3 bone material is good from the outside, but the internal bone consistency is fragile due to the complete dissolution of collagen. We therefore refrained from the application of ^14^C age analysis. Rather, we conducted ^230^Th/U-analysis of a flowstone encrusting and embedding phalangeal bones of the Chan Hol 3 skeleton (Fig 2). In a previous study, open-system behavior of U isotopes has been observed in speleothem calcite within 2 cm distance to the bone material [5]. To avoid similarly affected material, we therefore sampled at sufficient distance of > 2 cm to the bone material (Fig 2). The analysis yielded low U concentrations of 140 to 157 ng/g and low ^232^Th concentrations of 0.59 to 1.31 ng/g (Table 1). The U-isotopic composition, i.e. the ^234^U/^238^U activity ratio (expressed as ‰-deviation from secular radioactive equilibrium δ^234^U), is moderately positive and identical in all three samples with 37.1±1.1 ‰. To estimate the influence of initial ^230^Th on the ^230^Th/U age, e.g. by detrital contamination, our first order model is based on the conventionally used bulk Earth (^230^Th/^232^Th) activity ratio of 0.76±0.2, which further assumes radioactive equilibrium within the U-series decay chain. Using this assumption, calculated ages range from 10.35±0.2 ky BP to 10.87±0.22 ky BP, with uncertainties quoted as 2σ uncertainty of the mean (Table 1). The reference year for all ages given in the study is 1950 AD.

**Table 1:** ^230^Th/U measurements of the flowstone crust embedding a phalange of the Chan Hol skeleton.

N°	^238^U (ng/g)	^232^Th (ng/g)	^230^Th/^238^U (Act. rat.)	^230^Th/^232^Th (Act. rat.)	δ^234^U corr. (‰)	Age uncorr. (ky)	Age* corr. (ky)	Age** corr. (ky)	δ^234^U_initial_ (‰)	Depth [mm]
9770	139.862±0.040	0.8527±0.0026	0.0969±0.0013	49.06±0.68	35.23±2.47	10.66±0.15	10.50±0.17	**9.83±0.16**	36.3±2.5	3.5
9771	157.01±0.11	1.3132±0.0043	0.1007±0.0016	37.13±0.59	36.77±2.47	11.08±0.18	10.87±0.22	**9.94±0.18**	37.9±2.5	4.5
9769	144.952±0.024	0.5874±0.0053	0.0952±0.0016	72.29±1.41	36.14±2.28	10.45±0.21	10.35±0.20	**9.90±0.19**	37.2±2.3	5.5

Given the minor traces of ^232^Th (< 1.3 ng), the conventional detrital corrections are small (< 200 years) and lie within the uncertainty of the uncorrected ages. Ages are identical within 2σ uncertainty, except for the central sample which shows a moderate age inversion of 360 to 520 years outside the 2σ uncertainty of individual ages of 220 years. For stratigraphic reasons, we tested the assumption that this age inversion is related to an elevated ^230^Th/^232^Th activity ratio of the contaminating non-carbonate material, or an additional source of seepage water-derived ^230^Th. For this purpose, we presumed the three samples as coeval within an uncertainty of ±200 years; this assumption is based on the short period of carbonate growth (~520 years) and allows us to test the detrital ^230^Th model using an Osmond isochron [46]. From the Osmond isochron (S1 Fig) a contaminating ^230^Th/^232^Th activity ratio of 3.96±0.2 can be estimated leading to significant age corrections of up to -1100 years for the central-most contaminated carbonate. Assuming such a high ^230^Th contribution from cave seepage water or non-carbonate contamination leads to an average age of the three samples of 9.9±0.1 ky BP with no remaining age inversion, thus representing a minimum age of the carbonate. Given the small number of analyses, it remains difficult to privilege one over the other correction models. Consequently, we suggest that the layered carbonate embedding of the already present skeletal remains formed between 9.9±0.1 and 10.57±0.11 ky BP, reflecting the weighted mean age of both ^232^Th-correction models.

See Fig 2 for the position of samples. Errors are 2σ analytical errors. Corrected ^230^Th ages assume (1) an initial (^230^Th/^232^Th) activity ratio of 0.76 ± 0.2 (*); and (2) 3.96 ± 0.2 (**) derived from the Osmond isochrones approach assuming the three sub-samples as coeval.

### 4.6. Strontium Isotope analysis

The normalized ^87^Sr/^86^Sr ratio for the third right molar of Chan Hol 3 is 0.708878±0.000042 (2σ) and is given together with the SRM-987 standard values in S6 Table. Previous studies have mapped the Sr-isotopic composition of modern local fauna samples (e.g. mice, deer, peccary, rock, water, soils) and show increasing ^87^Sr/^86^Sr ratios from 0.704–0.706 in the southern part, to higher ratios of 0.707–0.709 in the northern part of the YP (e.g. [47–49]). The highest ratios (0.711–0.712) in Mesoamerica are associated with old plutonic and metamorphic rocks in the Maya Mountains of Belize [49]. Hodell et al. [49] grouped the strontium baseline values in five clusters that broadly match major geologic provinces (S2 Fig). Our measurement is well in the range of values expected for the northern YP and corresponds to cluster 1 defined by Hodell et al. [49] (S2 Fig).

## 5. Discussion

### 5.1. The Chan Hol 3 site

The Chan Hol 3 human skeletal remains were spread around a limestone slab, which nevertheless was not covered by bones, neither were bones detected by us below this rock (Fig 1C). The limestone is inclined by about 15° from the horizontal as it is positioned on top of a horizontally lying stalagmite (Fig 1E). We tentatively suggest that the flat limestone was intentionally placed by humans, perhaps serving as a “head rest” for the Chan Hol 3 individual. This interpretation is based on the following pieces of circumstantial evidence: the rock shelf is lithologically identical to other limestone slabs that cover the cave floor forming an *in situ* broken layer, but it partially overlies smaller fragments of this rock as well a broken stalagmite which also appears to be out of place. Transport of the heavy limestone slab and its elevated position above the cave floor are difficult to explain by water transport, nor does the slab correspond to limestone from the ceiling that would have fallen to the ground, there knocking down the stalagmite below the slab. As no human bones were detected below the limestone shelf, the skeleton must be younger than the placement of the slab.

Some bones were water-transported from their original position. While the mandible and long bones only moved down gravitationally and were slightly moved by the water, the round and air-filled skull was water-transported and rolled for about half a meter, where it rotated upside down (Fig 1E). The original position of the skeleton is therefore difficult to determine, but we tentatively suggest that it was preserved in a dorsal position.

At the time of decay and skeletonization, the cave was still dry and the Chan Hol 3 individual completely exposed on the cave floor. Water dripping from the cave ceiling resulted in the deposition of flowstone encrusting some of the bones, e.g. a solid calcite crust of up to 10 mm thickness covered the phalanges and fragmentary distal forearm epiphyses (Fig 2). From the shape of this flowstone it appears that the cave floor was sloping, so that the bones caused the water to be dammed. The flow around the bones caused those to be encrusted and resulted in a nearly laminar layered crust. Below these bones, a porous tufa-like flowstone is present.

Millimeter-thin crusts of calcite crystals covered several other bones, e.g. metacarpals, ulna, radius, maxillae. These crusts on the upward-directed surfaces of the long bones are caused by humidity and vapor within a dry cave. The lower cave floor-directed portion of these bones lack the crusts. Desiccation cracks on the cave floor further suggest a dry cave environment alternating with episodes of precipitation.

### 5.2. Skeleton age assessment

Based on the ^230^Th/U analyses of the layered flowstone crust overlying the phalangeal bones (Figs 2 and 7) the minimum *terminus ante quem* of the Chan Hol 3 skeleton is 9.9±0.1 ky BP.

Tufa-like flowstone immediately underlying the bone was not dated due to its porous texture and due to the potential effects of open-system behavior by U diffusion (e.g. [5]). In consequence, no data are available to define the amount of time that elapsed between the death of the individual and initial growth of the overlying carbonate, nor the time lapse needed for maceration and decay of this individual. Consequently, the precise age of the Chan Hol 3 skeleton within the Pleistocene-Holocene transition remains uncertain.

Clearly, flooding of the Chan Hol 3 site was significantly later, during the final middle Holocene rise of sea-level. This interpretation is supported by numerous charcoal concentrations of cultural origin located at various sites of the Chan Hol cave system in depth levels equivalent to those of the Chan Hol 3 site. These charcoal hearths ^14^C-dated to between 8,110±28 ^14^C y BP (9,122–8,999 cal y BP) and 7,177±27 ^14^C y BP (8,027–7,951 cal y BP) are therefore younger than both the Chan Hol 2 and 3 individuals [3, 6]. The Chan Hol 1 skeleton has been dated to 9,589±49 ^14^C y BP (11,073–10,817 cal y BP) [3, 6].

### 5.3. Assignation of the Chan Hol 3 individual

We originally expected that the skeleton documented here as Chan Hol 3 would have constituted the Chan Hol 2 skeleton, discovered in 2012 at only 140 m distance north of the one described here but stolen by unknown cave divers a few weeks after discovery [5]. However, after a closer inspection and comparison of the present osteological material from the Chan Hol 3 locality with photos taken prior to the looting of the Chan Hol 2 site, we are now positive that the two must represent different individuals. This interpretation results from the fact that several bones present in the material documented from Chan Hol 2 by Stinnesbeck et al. [5], are also present at Chan Hol 3. Among this replicated material is a complete mandible at Chan Hol 3, but fragmentary mandible at Chan Hol 2, upper lateral incisors, and complete right and left femora in both skeletons. In addition, two perforations are clearly seen in the right parietal of the Chan Hol 3 cranium, which are absent in the one documented for Chan Hol 2. Even though the prominent chin of the Chan Hol 3 mandible is usually considered to be a male feature, the femur size along with the gracile skull indicates a female individual, whereas Chan Hol 2 has been interpreted as a male [5].

### 5.4. Pathologies

Chan Hol 3 represents the fourth early female skeleton from the submerged caves of Tulum, together with the Naharon, Las Palmas and Hoyo Negro individuals. The right and left femora show strong anterior convexity (Fig 3L and 3M) which suggests high mobility, a feature often seen in hunter-gatherer populations [50]. Arthritis has been documented in the Chan Hol 3 skeleton, as in those from Hoyo Negro, Naharon, Muknal, Las Palmas and El Templo [1, 3, 6].

#### 5.4.1. Traumas

The Chan Hol 3 female survived three potential cranial traumas. The one identified on the left parietal bone was caused by a potential blunt impact, leading to a rounded opening of 2 mm diameter (Figs 3E and 6A). The cracks spread out in a circular manner around the impact and are due to a strong swelling. The second and third trauma on the parietal and occipital were caused by a hit, or heavy blow, on the back of the head with a sharp object (Figs 3C, 3E, 3F, 6A and 6B). The turning angle of 15° indicates a lateral impact. However, these traumas show signs of healing, suggesting that the Chan Hol 3 female survived all three.

#### 5.4.2. Potential treponemal bacterial disease

In addition to traumas, the skull of the Chan Hol 3 female exhibits irregular dents and crater-like deformations on the posterior parietal and occipital bones of the cranium. These are here interpreted as evidence for an infection (Fig 3F). The argument for infection is based on the interaction of these deformations with the lambdoidal suture. A taphonomical issue of bone preservation can be excluded, as neither the facial area of the skull, the mandible, nor postcranial elements, are affected by this form of bone alteration. Furthermore, the osteological remains of Chan Hol 1 and 2 from the same cave system, as also all other human and megafauna [51] remains from nearby caves in the area, are extremely well preserved and their bone surfaces are smooth. They allow for a reliable comparison of bone preservation levels based on the different find localities and from both fresh- and salt-water (e.g. Chan Hol 1 to 3 skeletons were contained in fresh-water, most other sites are salt-water). This excludes disintegration of bones by chemical reaction, producing holes, as seen for example on the dorsal surface of the cranium of the ground sloth *Xibalbaonyx* from the El Zapote cenote [51]. However, even at El Zapote where bones are extremely fragile due to heavy dissolution, the bone texture and surface are not deformed. Different to these bones collected from salt-water, the cortex of the Chan Hol 3 cranium is thick, especially the occipital area, indicating the absence of chemical dissolution. The morphological deformation on the skull in the Chan Hol 3 skeleton is more than likely to be a pathological pattern and not a preservation issue.

Pathological skull deformation as seen in the Chan Hol 3 female has not been documented for other early skeletons in the area and we here propose that it is possibly related to a treponemal bacterial disease (*Treponema peritonitis)* with subsequent osteitis/periostitis. The evolutionary history of treponemal diseases includes the origin of syphilis and is therefore the subject of on-going debate. As this would be the first evidence for *Treponema peritonitis* in an early Holocene skeleton in the New World, the Chan Hol 3 skeleton may potentially help to settle the debate. Today, the only other cases of these lesions in pre-Columbian skeletons are associated with the Atacameña culture of Chile, Argentina and southern Bolivia, from a specimen dating to 1650 BP [44], and a second dating to 2160 BP [52]. At this current stage, it is impossible to say which came first, the trauma to the skull or the bacterial infection. However, it makes more sense to say that the bacterial infection is a result of the trauma seen on the skull.

#### 5.4.3. Dental diseases

All pre-Mayan skeletons discovered to date in caves of the Tulum area present extraordinary light dental attrition for a hunter and gatherer society, but extremely high percentages of caries, aggressive periodontal diseases and dental abscesses (e.g. 53% of teeth affected in the 16–18 years old Naia female [53], but also see [3, 4, 54]. This pathological pattern is also evident in the Chan Hol 3 female that lost most of her teeth during life, and the remaining few show occlusal caries extending into the pulp cavity. Dental caries is an oral infectious bacterial disease, causing demineralization of the enamel and underlying dentine [55]. The disease is multifactorial, but usually related to buccal microflora, enamel composition, dental surface irregularities and fluorine contents of the ground water [56–58], as well as diet (e.g. carbon hydrate and sugar consumption) and body immunological responses [56–58]. Dental caries and abscess prevalence in the Yucatan skeletons are thus significantly higher than observed among other hunter-gatherers, except for a series of early Holocene skeletal remains from Lagoa Santa, Brazil [59]. The unexpected record of poor oral health, especially among females in this tropical South American locality, was attributed to a diet based on a highly cariogenic combination of wild tubers and fruits [59]. Elsewhere, caries is rare in coeval Paleoamerican skeletons of hunter-gatherer societies. In these latter individuals, e.g. from the Basin of Mexico, strong crown attrition is attributed to the consumption of hard fibrous foods ([2]; OTHERS), as is also interpreted for coeval groups from Europe [60]. Attrition is surprisingly light in the Chan Hol 3 female, but also in all other pre-Mayan skeletons known to date from the Tulum area, including Naia [53].

We therefore hypothesize that the paleodiet of the Tulum pre-Mayan humans must have differed significantly from that of other late Paleolithic hunter-gatherer societies, e.g. from Europe and the Basin of Mexico. We agree with the interpretation of Cucina et al. [53] that the Yucatan group depended on a nonabrasive diet that was at least seasonally rich in carbohydrates. As there is no evidence for early Holocene cultivation of plants on the YP, the unusually high amount of caries observed in the Tulum skeletons suggests a high consumption of tubercles and sweet (maltodextrine and sugar-rich) fruits, sweet cactus fruits, or honey from native stingless bees (*Meliposa* sp.) as part of the daily diet.

### 5.5. Provenance of early Yucatan settlers

The isotopic composition of tooth enamel is based on food and liquids consumed during infancy and does not change chemically during the life of the individual, and rather little during death. Therefore, the tooth enamel composition measured here should provide a fingerprint on the provenance (place of birth/youth) of the individual [48, 61, 62]. The value of ^87^Sr/^86^Sr of 0.708878 is close to the ones previously determined for areas located within 40 km distance to Tulum (0.7087 to 0.7091) [49, 63] and is also close to the one of modern seawater. Nevertheless, this value also fits to localities in the Northwestern part of the peninsula where identical values of 0.7089 to 0.7087 have repeatedly been observed [47, 49]. Consequently, we cannot say whether the female studied here continuously lived in the Tulum area, or whether she spent part of her life (esp. during adolescence) in the northwestern part of the peninsula.

### 5.6. Usage of the Chan Hol cave

Interpretation of the Chan Hol 3 site as a burial, as previously documented for Las Palmas, Muknal and Naharon skeletons [3, 4, 6], is inconclusive, even though the head and torso of the female individual may have been intentionally placed on a limestone slab moved from its original position. As in the Chan Hol 1 and 2 individuals, the legs of the Chan Hol 3 skeleton were outstretched, suggesting that the individual was lying on its back. However, the anatomical position is difficult to assess, since the female torso was disintegrated.

The *terminus ante quem* age of 9.9±0.1 ky of the Chan Hol 3 skeleton provides new supporting evidence for an early human use of the caves in the Tulum area. Human visits to caves in the area, including Chan Hol Cave, started during the Late Pleistocene, as is indicated by ^14^C ages of bones and by ^230^Th/U dating of limestone crusts and stalagmites encrusting bones (e.g. Hoyo Negro, Naharon, Chan Hol 2) [1, 3, 5, 6]. Although the early human settlers may have used the cave system as a burial ground or cult place [3, 4, 6], there is no positive evidence yet to support this scenario for the three human skeletons discovered at Chan Hol Cave. Rather, the death of the Chan Hol 1 to 3 individuals may have been accidental (e.g. treatment of the infection by trepanning) [3, 5]. Alternatively, the traumas discovered on the skull of the Chan Hol 3 female indicate potential personal violence. This interpretation is supported by the sharp incised (healing) wounds, rather than crushing or blunt force trauma which would have been caused by more rugged and larger objects (e.g. falling debris). In any case, it appears unlikely that visits to the Tulum cave system were routine procedures that were executed frequently.

### 5.7. Cranial morphology studies and the settlement of the Americas

The Chan Hol 3 woman has a mesocranial morphology (Fig 4; S5 Table), characterized by a flat forehead with wide cheekbones. These morphological traits have been detected in all pre-Mayan skeletons discovered in the Tulum cave system [1, 3–6] (Fig 1A). Their cranial morphology thus differs significantly from that of coeval Paleoindian skeletons from Central Mexico, e.g. Peñon III Woman, Tlapacoya Man, Metro Man, Chimalhuacan Man [3], which have been dated to between 12 to 7 ky BP (S1 Table) and all present dolicocephalic morphologies [3] (Figs 4 and 5; S5 Table). Based on these important morphometric differences among the skulls from Central Mexico and Tulum we suggest that at least two morphologically different Paleoindian human groups inhabited Mexico during the late Pleistocene-Early Holocene.

There are two potential hypotheses to explain the origin of these two different human groups: (1) They are derived from human populations from different geographical points of origin, or (2) They are the result of local micro-evolutionary processes such as genetic isolation, habitat preference, survival strategies, or even diet, that may have resulted in an *in situ* differentiation of the mesocranial morphologies identified in the skeletons of the Tulum area. They may thus have been a substantial factor in the evolutionary development of the subsequent Mayan populations from Mesoamerica. Clearly, however, the two scenarios are exclusively based on the morphometric aspects of these two populations and does not refer to their phylogenetic relationships, or genetic data. Full genome data from these populations is required to decide which hypothesis is correct because there are currently no data available.

## 6. Conclusions

The pre-Mayan skeleton here described from the Chan Hol cave near Tulum, Mexico, belongs to a woman of about 30 years with three severe cranial traumas, in addition to evidence for a possible treponemal bacterial disease. As seen in two other skeletons previously found in the same cave system, the new Chan Hol 3 woman decayed *in situ* at times when these shallow parts of the cave were still dry. After skeletonization, some of the bones were cemented to the cave floor by flowstone precipitated from calcite-saturated water (e.g. phalanges), while others were water-transported and dispersed over a small area of three square meters, either during heavy precipitation events or during the middle Holocene flooding of the cave. ^230^Th/U dating of the calcite crusts overlying a phalange indicate a minimum age of the skeleton of 9.9±0.1 ky BP.

The ten ate Pleistocene-Early Holocene individuals discovered and described so far in the submerged caves of Tulum, Yucatán, indicate a mobile group, eating sugary foods as indicated by the ubiquitous presence of caries. Their mesocranial skull morphologies are different to the dolicocephalic morphologies found in equivalent Paleoindian age human populations from Central Mexico that had strong teeth attrition indicating the consumption of hard foods. Our data thus support the presence of two morphologically different human groups with different subsistence strategies in Mexico during the Pleistocene-Holocene boundary transition.

## Supporting information

S1 Fig Osmond Isochron, assuming the three samples as coeval. This allows to test the detrital ^230^Th model [46]. The slope of the regression line yields a (^230^Th/^232^Th) activity ratio of the contaminating non-carbonate material of 3.96 ±0.2.
(TIF)

S2 Fig Simplified geologic map of the Maya region showing the age of exposed bedrock and results of cluster analysis based on ^87^Sr/^86^Sr measurements of water, bedrock, soils and plant [49]. Yellow star shows position of the Chan Hol cave and skeleton. Slightly modified figure taken from Hodell et al. [49].
(TIF)

S1 Table Radiometric dating of human bones, speleothem and charcoal associated with early skeletons from submerged caves in the Tulum area and from other North- and South America sites. (PDF)

S2 Table Osteometric measurements taken according to Buikstra and Ubelaker [17] and Howells [16]. Abbreviations are explained there.
(PDF)

S3 Table Breakdown of osteometric variables and cranial samples used for the PCA. Summary of Chronologies and Location of each Sample used for the PCA.
(PDF)

S4 Table The PC loadings for the first two Principal Components extracted from the PCA. For definition of the cranial variables see S3 Table.
(PDF)

S5 Table Cranial index and Superior Facial Index of skeletons from Mexico, North and South America presented in Fig 4. (PDF)

S6 Table Results of ^87^Sr/^86^Sr analyses of tooth enamel of the third left mandibular molar of the Chan Hol 3 skeleton found in the submerged Chan Hol cave at Tulum, Quintana Roo, Mexico. The data are normalized to standard SRM987.
(PDF)
