# Supplementary figures and images for: New evidence for an early settlement of the Yucatán Peninsula, Mexico: The Chan Hol 3 woman and her meaning for the Peopling of the Americas
Source: PLoS One. 2020 Feb 5;15(2):e0227984. doi: 10.1371/journal.pone.0227984 (PMC7001910; doi:10.1371/journal.pone.0227984)

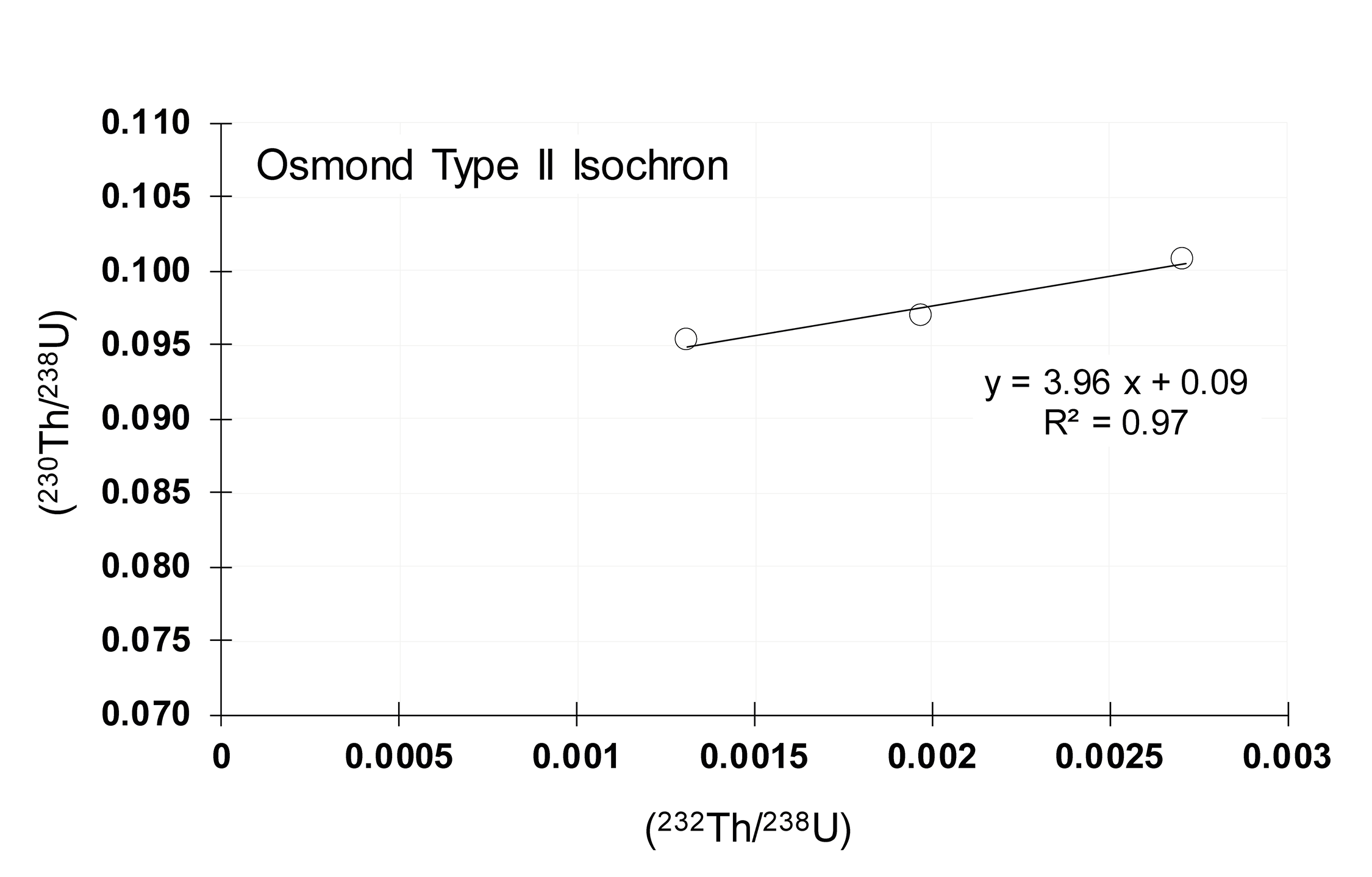

Supplement: S1 Fig — This allows to test the detrital 230Th model [46]. The slope of the regression line yields a (230Th/232Th) activity ratio of the contaminating non-carbonate material of 3.96 ±0.2. (TIF) [file pone.0227984.s001.tif]

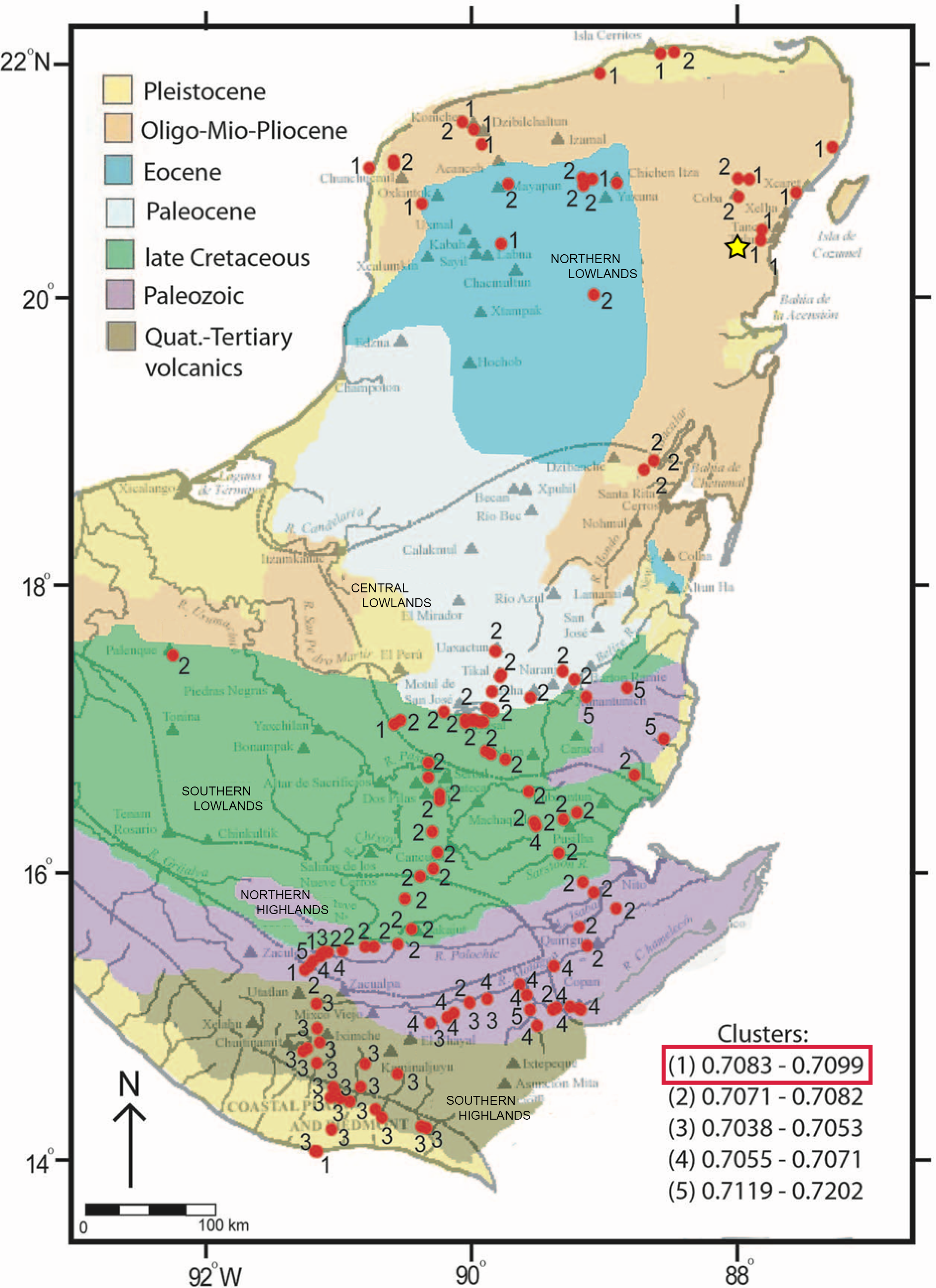

Supplement: S2 Fig — Yellow star shows position of the Chan Hol cave and skeleton. Slightly modified figure taken from Hodell et al. [49]. (TIF) [file pone.0227984.s002.tif]
