# Supplementary material for: New evidence for an early settlement of the Yucatán Peninsula, Mexico: The Chan Hol 3 woman and her meaning for the Peopling of the Americas
Source: PLoS One. 2020 Feb 5;15(2):e0227984. doi: 10.1371/journal.pone.0227984 (PMC7001910; doi:10.1371/journal.pone.0227984)
Supplement: S1 Table — (PDF) [file pone.0227984.s003.pdf]

**Skeleton**	**Location**	**Method**	**uncal BP**	**Age range/ cal BP**	**Reference**
Anzick 1	Montana, USA	^{14}C	10,680 ± 50	12,707–12,556	[62]
Chan Hol I	Quintana Roo, Mexico	^{14}C	9,589 ± 49	11,073-10,817	[3]
Chan Hol II	Quintana Roo, Mexico	$^{230}Th/U$	Approx. 13,000; >11,311 ± 370		[5]
Chan Hol III	Quintana Roo, Mexico	$^{230}Th/U$	>9,900		This article
Hoyo Negro	Quintana Roo, Mexico	^{14}C	10,976 ± 20	12,910-11,750	[1]
Lapa Vermelha IV	Lagoa Santa, Brazil	^{14}C	10,030 ± 60	11,710-11,243	[63]
Muknal	Quintana Roo, Mexico	^{14}C	8,890 ± 100	10,298-9,732-	[3, 4]

Naharon	Quintana Roo, Mexico	^{14}C	11,570 ± 65	13,571-13,337	[3]
Las Palmas	Quintana Roo, Mexico	^{14}C	8,050 ± 130	9,140-8,734	[3]
Peñon III	Mexico City, Mexico	^{14}C	10,755 ± 75	12,770–12,560	[3]
El Pit	Quintana Roo, Mexico	^{14}C	11,332 ± 64	13,340-13,229	[3]
El Templo	Quintana Roo, Mexico	^{14}C	9,589 ± 49	11,070-10,817	[3]
Tlapacoya I	Mexico City, Mexico	^{14}C	10,200 ± 65	12,150–11,610	[2]
