# Supplementary material for: New evidence for an early settlement of the Yucatán Peninsula, Mexico: The Chan Hol 3 woman and her meaning for the Peopling of the Americas
Source: PLoS One. 2020 Feb 5;15(2):e0227984. doi: 10.1371/journal.pone.0227984 (PMC7001910; doi:10.1371/journal.pone.0227984)
Supplement: S2 Table — Abbreviations are explained there. (PDF) [file pone.0227984.s004.pdf]

Craniometric Measurement (mm)	
GOL	170
XCB	130
BBH	125
XFB	-
ZYB	123
NPH	63
BPL	95
BNL	100
NOL	168
FRC	100
PAC	132
OCC	115
NLH	52

NLB	25
OBB	37
OBH	35
DKB	22
**Femoral Measurement (mm)**	
Femoral Head Diameter	39
Mx Fem Length	430
