# Supplementary material for: New evidence for an early settlement of the Yucatán Peninsula, Mexico: The Chan Hol 3 woman and her meaning for the Peopling of the Americas
Source: PLoS One. 2020 Feb 5;15(2):e0227984. doi: 10.1371/journal.pone.0227984 (PMC7001910; doi:10.1371/journal.pone.0227984)
Supplement: S3 Table — Summary of Chronologies and Location of each Sample used for the PCA. (PDF) [file pone.0227984.s005.pdf]

Sample	Date	Males	Females	Total	Source
Archaic Colombia	5-3 ky	7	13	20	[25]
Arikara	0.4-0.1 ky	42	27	69	[16, 21-23]
Californian Coast	5-4 ky	14	4	18	Authors
Central Mexico	12-7 ky	2	3	5	[3]
Coahuila	1 ky	44	37	81	[27]
Lagoa Santa	12-7 ky	18	11	29	[26]
Paleo Colombia	11-6 ky	6	8	14	[25]
Peru	1-0.5 ky	55	55	110	[16, 21-23]
Santa Cruz	0.4 ky	51	51	102	[16, 21-23]
Yucatan	13-9 ky	2	2	4	[28 & Authors]
**Total**		**241**	**211**	**452**	

Cranial Variables	
GOL	Max. Cranial Length
XCB	Max. Cranial Breadth
BBH	Basion-Bregma Height
XFB	Maximum Frontal Breadth
ZYB	Bizygomatic Breadth
NPH	Nasion-Prosthion Height
NOL	Nasio-Occipital Length
FRC	Frontal Chord
NLH	Nasal Height
NLB	Nasal Breadth
OBB	Orbital Breadth
OBH	Orbital Height

SpecNo	OldNo	Location	PopCode	Age	Sex	SexCode	GOL	XCB	BBH	XFB	ZYB	NPH	NOL	FRC	NLH	NLB	OBB	OBH
Co1	1	Coahuila (1ky)	1	Adult	Male	1	173	137	139	119	135	72	140	111	55	26	42.5	36.5
Co2	2	Coahuila (1ky)	1	Adult	Male	1	178	134	135	112	139	71	142	109	51	26	39	34.25
Co3	3	Coahuila (1ky)	1	Adult	Male	1	183	134	134	114	136	74	137	111	52	27	37.5	32.5
Co4	4	Coahuila (1ky)	1	Adult	Male	1	181	130	137	110	133	73	136	117	55	24.5	37.5	35.5
Co5	5	Coahuila (1ky)	1	Adult	Male	1	189	130	136	115	132	74	141	108	53	25.5	37.5	35.75
Co6	6	Coahuila (1ky)	1	Adult	Male	1	182	133	137	109	134	72	139	116	52	25	38	32.75
Co7	8	Coahuila (1ky)	1	Adult	Male	1	186	135	137	114	140	73	138	116	53	25	38.5	34.75
Co8	9	Coahuila (1ky)	1	Adult	Male	1	178	136	132	112	140	72	136	104	50	28.5	37	35.25
Co9	10	Coahuila (1ky)	1	Adult	Male	1	183	135	135	112	140	74	140	114	54.5	27.5	38.5	31.5
Co10	12	Coahuila (1ky)	1	Adult	Male	1	193	135	135	108	134	78	140	117	53	25	48.5	33.75

SpecNo	OldNo	Location	PopCode	Age	Sex	SexCode	GOL	XCB	BBH	XFB	ZYB	NPH	NOL	FRC	NLH	NLB	OBB	OBH
Co11	13	Coahuila (1ky)	1	Adult	Male	1	186	142	134	115	142	75	149	110	50	27.5	40	36.5
Co12	14	Coahuila (1ky)	1	Adult	Male	1	188	136	146	118	141	73	142	116	53.5	27	38.5	34.75
Co13	15	Coahuila (1ky)	1	Adult	Male	1	190	131	137	110	141	73	135	116	51	26	39.5	36.75
Co14	16	Coahuila (1ky)	1	Adult	Male	1	180	127	133	104	132	71	133	112	47.5	24.5	38	31
Co15	17	Coahuila (1ky)	1	Adult	Male	1	176	130	136	111	136	70.5	135	111	51	24.9	37.85	33.7
Co16	18	Coahuila (1ky)	1	Adult	Male	1	184	129	133	106	134	72.5	135	110	51	24	38	32.5
Co17	19	Coahuila (1ky)	1	Adult	Male	1	182	127	132	108	132	73.5	139	107	52	24	37.75	33.25
Co18	20	Coahuila (1ky)	1	Adult	Male	1	182	139	136	115	136	72	136	112	50	26.5	39	34.75
Co19	21	Coahuila (1ky)	1	Adult	Male	1	179	135	132	119	135	72	134	110	52	26	37	33.75
Co20	22	Coahuila (1ky)	1	Adult	Male	1	186	130	133	110	140	71	140	114	53	26	38.5	34.75
Co21	23	Coahuila (1ky)	1	Adult	Male	1	186	142	143	119	141	74	144	115	50.5	28.5	39.5	36.5
Co22	24	Coahuila (1ky)	1	Adult	Male	1	184	138	132	110	140	77	144	112	53.5	26	41.5	35.25
Co23	25	Coahuila (1ky)	1	Adult	Male	1	175	129	129	109	132	68	129	104	48	24	37.5	32
Co24	26	Coahuila (1ky)	1	Adult	Male	1	188	138	138	119	140	74	145	117	54	29	38.5	33.75
Co25	27	Coahuila (1ky)	1	Adult	Male	1	183	132	132	118	133	72	140	106	57	26.5	38	37.75
Co26	28	Coahuila (1ky)	1	Adult	Male	1	183	128	132	104	134	72	141	104	55	25	38	35.5
Co27	29	Coahuila (1ky)	1	Adult	Male	1	179	133	133	110	134	74	142	102	51	23.5	38	34.75
Co28	30	Coahuila (1ky)	1	Adult	Male	1	185	131	134	111	135	68	138	113	47	25	37.25	29.25
Co29	31	Coahuila (1ky)	1	Adult	Male	1	178	136	137	114	140	70	136	111	51	24	37.25	33.75
Co30	32	Coahuila (1ky)	1	Adult	Male	1	184	123	133	109	134	72	140	114	51	24	36.25	31.5
Co31	33	Coahuila (1ky)	1	Adult	Male	1	181	120	137	110	145	75	147	110	57.5	25	40.25	33.5
Co32	34	Coahuila (1ky)	1	Adult	Male	1	186	137	141	119	140	70	142	113	50	26	39.75	34
Co33	35	Coahuila (1ky)	1	Adult	Male	1	181	134	134	112	138	72.5	139	109	54	26.5	37.5	32.25
Co34	36	Coahuila (1ky)	1	Adult	Male	1	184	129	137	116	144	76	142	107	56.5	25	42.25	37.75
Co35	37	Coahuila (1ky)	1	Adult	Male	1	178	129	131	104	130	68	139	107	50	24	38	34.75
Co36	38	Coahuila (1ky)	1	Adult	Male	1	177	134	140	113	135	75	144	107	53.5	27.5	40	34.75
Co37	39	Coahuila (1ky)	1	Adult	Male	1	183	132	135	111	139	76	140	112	51	27	40.75	36
Co38	40	Coahuila (1ky)	1	Adult	Male	1	185	133	136	112	139	75	141	112	54	25	38	34.75
Co39	41	Coahuila (1ky)	1	Adult	Male	1	180	128	130	108	133	72.5	139	103	51	26	38	34.75
Co40	42	Coahuila (1ky)	1	Adult	Male	1	188	139	136	115	139	69	147	117	56	27	38.25	34.25
Co41	43	Coahuila (1ky)	1	Adult	Male	1	181	134	132	110	141	72	137	100	52	25	39.75	34.25
Co42	44	Coahuila (1ky)	1	Adult	Male	1	176	132	136	106	137	76	137	106	56	26	39	35.5
Co43	45	Coahuila (1ky)	1	Adult	Male	1	186	135	139	110	140	72	140	112	53	26	39.75	34.75
Co44	46	Coahuila (1ky)	1	Adult	Male	1	181	134	134	108	138	73	139	109	52	29.5	20	33.75
Co45	1	Coahuila (1ky)	1	Adult	Female	2	174	132	133	119	135	68	132	107	53	26.5	39.75	33.5
Co46	2	Coahuila (1ky)	1	Adult	Female	2	184	136	131	112	134	70	134	111	48.5	26	40.5	33
Co47	3	Coahuila (1ky)	1	Adult	Female	2	181	128	129	107	134	67	140	105	42	28	38.25	32
Co48	4	Coahuila (1ky)	1	Adult	Female	2	180	130	135	107	133	72	136	110	49	25	37.5	33.75

SpecNo	OldNo	Location	PopCode	Age	Sex	SexCode	GOL	XCB	BBH	XFB	ZYB	NPH	NOL	FRC	NLH	NLB	OBB	OBH
Co49	5	Coahuila (1ky)	1	Adult	Female	2	170	130	133	109	126	70	129	99	46.5	24.5	37.5	35.5
Co50	6	Coahuila (1ky)	1	Adult	Female	2	182	138	134	112	135	70	140	109	53.5	27	38	35.25
Co51	7	Coahuila (1ky)	1	Adult	Female	2	182	122	124	102	124	70	132	106	49	26	37.75	34
Co52	8	Coahuila (1ky)	1	Adult	Female	2	180	134	127	113	133	70.5	122	108	48.5	25.5	40.5	36
Co53	9	Coahuila (1ky)	1	Adult	Female	2	171	128	134	107	132	63	134	105	47	25.5	39.5	34
Co54	10	Coahuila (1ky)	1	Adult	Female	2	179	130	129	106	130	71	132	108	49	23	38.25	35
Co55	13	Coahuila (1ky)	1	Adult	Female	2	176	126	123	108	127	69	136	108	50	23	36.25	33
Co56	14	Coahuila (1ky)	1	Adult	Female	2	180	133	138	114	125	67	142	112	50	23.5	38.5	36.5
Co57	15	Coahuila (1ky)	1	Adult	Female	2	181	128	123	108	132	68.5	134	109	51.5	27	40.25	39
Co58	16	Coahuila (1ky)	1	Adult	Female	2	182	137	137	115	135	72.5	136	115	51	26.5	39.5	36
Co59	17	Coahuila (1ky)	1	Adult	Female	2	176	124	135	103	128	68.5	130	104	49.5	25	38	35.5
Co60	18	Coahuila (1ky)	1	Adult	Female	2	176	129	134	109	129	73.5	132	107	53	26	37.75	35.5
Co61	19	Coahuila (1ky)	1	Adult	Female	2	185	130	132	108	133	70	136	109	46	24	18.25	33.25
Co62	20	Coahuila (1ky)	1	Adult	Female	2	179	133	123	109	130	65	130	105	49	25	36.75	33
Co63	21	Coahuila (1ky)	1	Adult	Female	2	188	132	128	111	128	63	132	110	48	24.5	36.5	33.5
Co64	22	Coahuila (1ky)	1	Adult	Female	2	176	126	129	107	128	64	136	99	48	24	38	33
Co65	23	Coahuila (1ky)	1	Adult	Female	2	169	127	126	102	126	70	131	102	51	24	36.75	34.5
Co66	24	Coahuila (1ky)	1	Adult	Female	2	174	124	123	105	120	61	138	105	44.5	24	35.5	32.25
Co67	25	Coahuila (1ky)	1	Adult	Female	2	181	136	130	113	128	69	134	111	49	28	37.5	33
Co68	26	Coahuila (1ky)	1	Adult	Female	2	171	128	121	103	129	65	136	101	47.5	25	40.25	33.5
Co69	27	Coahuila (1ky)	1	Adult	Female	2	172	129	132	106	124	69	134	104	51	24	37.5	32.75
Co70	28	Coahuila (1ky)	1	Adult	Female	2	178	135	130	110	128	67	134	102	49	25	37.5	33.75
Co71	29	Coahuila (1ky)	1	Adult	Female	2	174	127	130	104	128	68	132	109	47	24	39	34
Co72	30	Coahuila (1ky)	1	Adult	Female	2	186	125	123	106	122	66	131	100	50	26.5	38.5	33.75
Co73	31	Coahuila (1ky)	1	Adult	Female	2	173	130	132	109	128	70	136	108	51.5	24.5	38.75	33.25
Co74	32	Coahuila (1ky)	1	Adult	Female	2	174	131	131	102	129	66	132	104	47	22.5	39	34
Co75	33	Coahuila (1ky)	1	Adult	Female	2	167	129	127	106	128	70.5	133	103	52.5	25	40.5	34.5
Co76	34	Coahuila (1ky)	1	Adult	Female	2	172	132	134	110	127	68	136	104	49	24	38.5	35.75
Co77	35	Coahuila (1ky)	1	Adult	Female	2	168	125	125	105	123	65	123	104	45	24	35.75	33
Co78	36	Coahuila (1ky)	1	Adult	Female	2	179	129	129	107	125	69	131	105	50	25	36.75	34.5
Co79	37	Coahuila (1ky)	1	Adult	Female	2	173	128	129	110	132	70	141	107	50	27	38.5	37.5
Co80	38	Coahuila (1ky)	1	Adult	Female	2	175	129	134	111	132	71	126	111	48	27	37	33.75
Co81	39	Coahuila (1ky)	1	Adult	Female	2	180	130	129	108	128	68	132	109	48.5	24	38.5	35
Tlapacoya		Central Mexico (12 - 7 ky)	2	Adult	Female	2	197	135	132	109	140	66	136	129	49	26	39.5	36
C. d. Tecolote		Central Mexico (12 - 7 ky)	2	Adult	Male	1	204	141	134	106	140	58	140	119	43	26	40.58	36
Chimalhuacan		Central Mexico (12 - 7 ky)	2	Adult	Male	1	197	141	134	101	145	77	142	111	56	25	42	36
M. Balderas		Central Mexico (12 - 7 ky)	2	Adult	Female	2	196	140	134	106	140	66	136	111	49	26	45	38
Penon III		Central Mexico (12 - 7 ky)	2	Adult	Female	2	187	140	129	101	134	63	136	116	46	24	38	35

SpecNo	OldNo	Location	PopCode	Age	Sex	SexCode	GOL	XCB	BBH	XFB	ZYB	NPH	NOL	FRC	NLH	NLB	OBB	OBH
1399		Peru (1 - 0.5 ky)	3	Adult	Male	1	183	141	130	117	138	65	180	111	49	26	37	34
1400		Peru (1 - 0.5 ky)	3	Adult	Male	1	180	138	130	113	144	64	174	108	50	28	39	34
1401		Peru (1 - 0.5 ky)	3	Adult	Male	1	177	139	129	117	135	67	176	111	48	28	37	33
1402		Peru (1 - 0.5 ky)	3	Adult	Male	1	175	146	129	115	137	74	175	116	52	23	39	36
1404		Peru (1 - 0.5 ky)	3	Adult	Male	1	178	140	126	119	132	63	178	111	48	23	37	36
1405		Peru (1 - 0.5 ky)	3	Adult	Male	1	182	139	136	114	131	70	181	112	51	25	38	34
1406		Peru (1 - 0.5 ky)	3	Adult	Male	1	186	149	131	126	142	73	183	118	53	26	39	35
1407		Peru (1 - 0.5 ky)	3	Adult	Male	1	192	139	146	115	149	74	190	111	54	30	41	34
1408		Peru (1 - 0.5 ky)	3	Adult	Male	1	178	139	133	111	138	64	176	110	49	27	37	35
1409		Peru (1 - 0.5 ky)	3	Adult	Male	1	180	142	143	118	141	69	180	117	54	25	41	33
1411		Peru (1 - 0.5 ky)	3	Adult	Male	1	182	139	135	115	136	65	179	114	51	25	37	34
1412		Peru (1 - 0.5 ky)	3	Adult	Male	1	176	144	123	113	136	72	174	105	53	27	36	35
1413		Peru (1 - 0.5 ky)	3	Adult	Male	1	192	137	128	118	140	69	190	115	51	29	39	33
1415		Peru (1 - 0.5 ky)	3	Adult	Male	1	172	140	133	116	136	66	169	107	50	26	40	34
1416		Peru (1 - 0.5 ky)	3	Adult	Male	1	182	133	129	112	129	64	181	112	46	26	37	33
1417		Peru (1 - 0.5 ky)	3	Adult	Male	1	184	141	144	122	132	69	182	114	50	25	39	36
1419		Peru (1 - 0.5 ky)	3	Adult	Male	1	183	135	128	115	131	73	180	114	51	24	39	33
1420		Peru (1 - 0.5 ky)	3	Adult	Male	1	178	140	135	118	137	69	176	107	48	26	38	34
1421		Peru (1 - 0.5 ky)	3	Adult	Male	1	170	134	125	115	124	64	169	103	49	27	37	32
1423		Peru (1 - 0.5 ky)	3	Adult	Male	1	179	141	132	117	137	69	177	113	51	25	38	35
1424		Peru (1 - 0.5 ky)	3	Adult	Male	1	182	138	134	121	136	66	180	111	53	25	39	36
1425		Peru (1 - 0.5 ky)	3	Adult	Male	1	169	140	128	107	136	70	168	102	51	26	37	35
1427		Peru (1 - 0.5 ky)	3	Adult	Male	1	181	136	130	114	133	72	180	108	52	24	38	33
1428		Peru (1 - 0.5 ky)	3	Adult	Male	1	173	142	122	114	135	64	172	107	48	28	36	32
1429		Peru (1 - 0.5 ky)	3	Adult	Male	1	174	142	128	119	134	66	173	111	48	24	40	35
1431		Peru (1 - 0.5 ky)	3	Adult	Male	1	172	135	127	109	129	63	170	100	46	24	37	32
1432		Peru (1 - 0.5 ky)	3	Adult	Male	1	172	139	137	117	137	76	169	115	55	25	39	37
1433		Peru (1 - 0.5 ky)	3	Adult	Male	1	178	142	129	121	138	72	177	114	52	23	39	36
1434		Peru (1 - 0.5 ky)	3	Adult	Male	1	175	137	125	115	134	71	174	106	51	26	41	36
1436		Peru (1 - 0.5 ky)	3	Adult	Male	1	183	138	131	118	136	67	182	112	49	25	38	32
1437		Peru (1 - 0.5 ky)	3	Adult	Male	1	174	135	129	122	130	65	172	106	50	27	40	33
1439		Peru (1 - 0.5 ky)	3	Adult	Male	1	181	143	129	120	132	70	181	113	53	25	38	35
1440		Peru (1 - 0.5 ky)	3	Adult	Male	1	172	135	132	115	131	71	172	108	54	20	40	35
1441		Peru (1 - 0.5 ky)	3	Adult	Male	1	173	130	124	111	132	64	172	111	48	26	36	34
1443		Peru (1 - 0.5 ky)	3	Adult	Male	1	176	138	124	109	137	64	174	105	50	27	37	35
1444		Peru (1 - 0.5 ky)	3	Adult	Male	1	177	132	124	111	127	67	175	108	48	23	36	35
1445		Peru (1 - 0.5 ky)	3	Adult	Male	1	170	135	131	113	135	74	169	110	53	23	38	35
1447		Peru (1 - 0.5 ky)	3	Adult	Male	1	184	140	135	123	134	69	181	116	50	27	38	34

SpecNo	OldNo	Location	PopCode	Age	Sex	SexCode	GOL	XCB	BBH	XFB	ZYB	NPH	NOL	FRC	NLH	NLB	OBB	OBH
1448		Peru (1 - 0.5 ky)	3	Adult	Male	1	178	141	130	119	141	71	175	107	54	26	39	36
1449		Peru (1 - 0.5 ky)	3	Adult	Male	1	180	133	130	115	137	67	178	106	48	26	39	35
1451		Peru (1 - 0.5 ky)	3	Adult	Male	1	175	139	129	117	135	60	171	104	47	24	38	32
1452		Peru (1 - 0.5 ky)	3	Adult	Male	1	176	141	132	116	137	66	175	106	49	26	36	31
1453		Peru (1 - 0.5 ky)	3	Adult	Male	1	178	136	132	115	133	71	177	114	50	22	40	36
1454		Peru (1 - 0.5 ky)	3	Adult	Female	2	169	136	128	107	127	60	168	100	48	26	38	34
1455		Peru (1 - 0.5 ky)	3	Adult	Female	2	177	133	130	111	124	69	176	111	50	25	37	31
1456		Peru (1 - 0.5 ky)	3	Adult	Female	2	168	137	126	111	117	59	167	107	44	26	37	34
1457		Peru (1 - 0.5 ky)	3	Adult	Female	2	169	139	124	109	119	61	168	104	43	25	37	33
1458		Peru (1 - 0.5 ky)	3	Adult	Female	2	175	139	132	115	126	71	174	108	52	24	39	38
1460		Peru (1 - 0.5 ky)	3	Adult	Female	2	167	133	125	110	129	66	167	105	47	23	37	34
1462		Peru (1 - 0.5 ky)	3	Adult	Female	2	168	137	125	107	123	62	169	101	48	23	36	35
1463		Peru (1 - 0.5 ky)	3	Adult	Female	2	167	135	117	107	127	62	166	103	47	24	36	34
1464		Peru (1 - 0.5 ky)	3	Adult	Female	2	171	129	126	111	124	62	171	102	48	24	36	34
1466		Peru (1 - 0.5 ky)	3	Adult	Female	2	170	134	121	113	119	61	169	102	45	22	36	33
1467		Peru (1 - 0.5 ky)	3	Adult	Female	2	160	141	124	110	129	58	160	103	45	24	36	32
1468		Peru (1 - 0.5 ky)	3	Adult	Female	2	173	127	125	110	122	62	172	108	46	24	37	33
1470		Peru (1 - 0.5 ky)	3	Adult	Female	2	165	135	127	113	122	62	164	108	46	27	36	35
1471		Peru (1 - 0.5 ky)	3	Adult	Female	2	169	134	122	116	122	62	166	103	45	24	35	33
1472		Peru (1 - 0.5 ky)	3	Adult	Female	2	172	138	123	114	129	65	172	109	48	26	36	36
1474		Peru (1 - 0.5 ky)	3	Adult	Female	2	176	142	127	122	131	68	172	110	51	23	36	34
1475		Peru (1 - 0.5 ky)	3	Adult	Female	2	169	133	121	111	124	64	169	107	49	23	36	36
1476		Peru (1 - 0.5 ky)	3	Adult	Female	2	178	137	131	117	129	66	177	113	48	21	38	35
1478		Peru (1 - 0.5 ky)	3	Adult	Female	2	164	137	129	114	134	63	163	105	49	21	37	34
1479		Peru (1 - 0.5 ky)	3	Adult	Female	2	177	140	126	119	125	66	176	112	50	23	36	33
1480		Peru (1 - 0.5 ky)	3	Adult	Female	2	170	133	127	112	126	65	168	105	48	25	36	35
1482		Peru (1 - 0.5 ky)	3	Adult	Female	2	172	142	122	117	132	64	172	108	50	25	39	36
1483		Peru (1 - 0.5 ky)	3	Adult	Female	2	176	129	128	108	125	59	174	104	44	25	35	32
1484		Peru (1 - 0.5 ky)	3	Adult	Female	2	171	141	127	115	127	64	170	106	46	22	38	35
1486		Peru (1 - 0.5 ky)	3	Adult	Female	2	169	139	131	114	127	64	168	103	51	24	34	34
1487		Peru (1 - 0.5 ky)	3	Adult	Female	2	176	139	126	117	127	66	174	109	47	26	39	33
1488		Peru (1 - 0.5 ky)	3	Adult	Female	2	169	136	128	109	133	69	169	108	50	27	38	36
1490		Peru (1 - 0.5 ky)	3	Adult	Female	2	164	131	120	111	118	63	164	101	48	23	36	35
1491		Peru (1 - 0.5 ky)	3	Adult	Female	2	176	136	117	113	130	71	176	103	49	22	37	35
1492		Peru (1 - 0.5 ky)	3	Adult	Female	2	164	128	122	109	124	63	164	105	44	24	36	33
1494		Peru (1 - 0.5 ky)	3	Adult	Female	2	170	134	119	107	120	66	171	103	50	24	36	34
1495		Peru (1 - 0.5 ky)	3	Adult	Female	2	157	129	127	113	125	61	156	104	45	26	37	33
1496		Peru (1 - 0.5 ky)	3	Adult	Female	2	167	138	128	120	126	65	166	109	50	24	40	36

SpecNo	OldNo	Location	PopCode	Age	Sex	SexCode	GOL	XCB	BBH	XFB	ZYB	NPH	NOL	FRC	NLH	NLB	OBB	OBH
1498		Peru (1 - 0.5 ky)	3	Adult	Female	2	161	136	118	111	128	56	159	106	47	26	39	35
1499		Peru (1 - 0.5 ky)	3	Adult	Female	2	171	145	134	124	135	73	171	112	54	23	38	36
1500		Peru (1 - 0.5 ky)	3	Adult	Female	2	175	131	129	109	132	67	175	110	50	25	38	34
1502		Peru (1 - 0.5 ky)	3	Adult	Female	2	176	139	133	112	131	72	176	112	51	23	38	37
1503		Peru (1 - 0.5 ky)	3	Adult	Female	2	171	137	128	120	129	63	170	105	46	23	39	32
1504		Peru (1 - 0.5 ky)	3	Adult	Female	2	175	138	128	115	128	68	175	110	50	24	37	33
1506		Peru (1 - 0.5 ky)	3	Adult	Female	2	160	134	120	116	125	66	160	102	47	23	37	35
1507		Peru (1 - 0.5 ky)	3	Adult	Female	2	173	133	126	113	130	57	172	110	42	24	39	32
1508		Peru (1 - 0.5 ky)	3	Adult	Female	2	160	128	121	107	126	63	159	98	47	26	36	33
1724		Peru (1 - 0.5 ky)	3	Adult	Male	1	177	137	135	114	133	63	174	107	47	24	38	34
1725		Peru (1 - 0.5 ky)	3	Adult	Male	1	182	140	134	120	132	68	180	112	50	25	38	35
1726		Peru (1 - 0.5 ky)	3	Adult	Male	1	175	142	127	123	141	66	174	109	50	25	41	36
1727		Peru (1 - 0.5 ky)	3	Adult	Male	1	188	138	136	113	139	71	187	118	54	25	39	36
1728		Peru (1 - 0.5 ky)	3	Adult	Male	1	173	129	129	113	130	63	170	102	48	25	38	31
1729		Peru (1 - 0.5 ky)	3	Adult	Male	1	178	133	124	108	134	66	176	113	49	25	38	34
1730		Peru (1 - 0.5 ky)	3	Adult	Male	1	178	138	137	113	133	66	176	114	50	26	37	36
1731		Peru (1 - 0.5 ky)	3	Adult	Male	1	182	133	135	112	133	67	180	111	50	24	40	33
1732		Peru (1 - 0.5 ky)	3	Adult	Male	1	173	130	132	107	136	70	171	108	52	23	40	36
1733		Peru (1 - 0.5 ky)	3	Adult	Male	1	168	136	129	113	133	63	165	105	48	23	37	34
1734		Peru (1 - 0.5 ky)	3	Adult	Male	1	176	133	121	108	132	67	174	101	52	24	36	32
1735		Peru (1 - 0.5 ky)	3	Adult	Male	1	176	135	123	107	131	69	176	106	52	26	38	35
1736		Peru (1 - 0.5 ky)	3	Adult	Female	2	158	130	120	108	119	62	158	96	47	22	35	35
1737		Peru (1 - 0.5 ky)	3	Adult	Female	2	164	133	129	110	123	62	164	101	46	23	36	33
1738		Peru (1 - 0.5 ky)	3	Adult	Female	2	166	126	120	105	126	63	165	98	48	28	37	35
1739		Peru (1 - 0.5 ky)	3	Adult	Female	2	166	135	125	112	125	57	164	103	43	24	36	33
1740		Peru (1 - 0.5 ky)	3	Adult	Female	2	172	139	127	115	126	64	170	108	48	22	37	34
1741		Peru (1 - 0.5 ky)	3	Adult	Female	2	169	137	124	115	124	61	167	104	49	25	36	33
1742		Peru (1 - 0.5 ky)	3	Adult	Female	2	168	134	125	107	122	64	166	104	49	22	37	34
1743		Peru (1 - 0.5 ky)	3	Adult	Female	2	166	131	121	107	123	62	165	104	51	25	36	34
1744		Peru (1 - 0.5 ky)	3	Adult	Female	2	168	130	123	111	124	64	165	99	49	24	38	35
1745		Peru (1 - 0.5 ky)	3	Adult	Female	2	174	136	125	110	124	60	174	102	49	23	37	35
1746		Peru (1 - 0.5 ky)	3	Adult	Female	2	161	137	120	112	123	61	160	99	46	22	36	36
1747		Peru (1 - 0.5 ky)	3	Adult	Female	2	168	138	122	108	123	65	168	105	47	21	36	33
1748		Peru (1 - 0.5 ky)	3	Adult	Female	2	168	123	121	105	120	62	166	102	44	25	34	33
1796		Arikara (0.4 - 0.1 ky)	4	Adult	Male	1	178	140	131	112	133	71	177	108	52	24	38	34
1797		Arikara (0.4 - 0.1 ky)	4	Adult	Male	1	172	136	131	114	140	71	171	107	53	26	41	34
1798		Arikara (0.4 - 0.1 ky)	4	Adult	Male	1	188	137	130	113	140	69	186	111	54	25	41	33
1799		Arikara (0.4 - 0.1 ky)	4	Adult	Male	1	180	144	133	117	145	72	178	105	54	26	41	35

SpecNo	OldNo	Location	PopCode	Age	Sex	SexCode	GOL	XCB	BBH	XFB	ZYB	NPH	NOL	FRC	NLH	NLB	OBB	OBH
1800		Arikara (0.4 - 0.1 ky)	4	Adult	Male	1	182	135	134	115	141	75	181	109	55	29	40	34
1801		Arikara (0.4 - 0.1 ky)	4	Adult	Male	1	186	143	131	116	139	76	183	113	56	27	40	37
1802		Arikara (0.4 - 0.1 ky)	4	Adult	Male	1	176	141	127	121	140	70	175	106	55	29	40	34
1803		Arikara (0.4 - 0.1 ky)	4	Adult	Male	1	173	141	134	115	138	71	172	107	54	25	39	33
1804		Arikara (0.4 - 0.1 ky)	4	Adult	Male	1	185	147	141	120	143	80	184	115	61	31	42	37
1805		Arikara (0.4 - 0.1 ky)	4	Adult	Male	1	175	152	136	123	147	74	173	109	58	28	42	37
1806		Arikara (0.4 - 0.1 ky)	4	Adult	Male	1	175	153	138	128	151	79	172	113	56	26	41	36
1807		Arikara (0.4 - 0.1 ky)	4	Adult	Male	1	176	139	132	117	139	71	175	105	55	27	41	37
1808		Arikara (0.4 - 0.1 ky)	4	Adult	Male	1	177	142	132	113	146	71	175	106	55	26	40	32
1809		Arikara (0.4 - 0.1 ky)	4	Adult	Male	1	189	148	135	122	146	77	188	113	55	30	42	36
1810		Arikara (0.4 - 0.1 ky)	4	Adult	Male	1	181	146	136	118	150	72	177	111	56	29	40	36
1811		Arikara (0.4 - 0.1 ky)	4	Adult	Male	1	167	139	132	119	138	66	166	103	52	26	42	34
1812		Arikara (0.4 - 0.1 ky)	4	Adult	Male	1	182	133	134	113	141	73	181	114	56	28	40	34
1813		Arikara (0.4 - 0.1 ky)	4	Adult	Male	1	175	139	133	113	138	69	174	105	55	27	40	35
1814		Arikara (0.4 - 0.1 ky)	4	Adult	Male	1	177	147	132	121	142	72	175	109	55	27	39	36
1815		Arikara (0.4 - 0.1 ky)	4	Adult	Male	1	184	143	134	111	139	65	182	109	50	27	40	36
1816		Arikara (0.4 - 0.1 ky)	4	Adult	Male	1	178	141	136	117	140	70	177	107	56	29	40	36
1817		Arikara (0.4 - 0.1 ky)	4	Adult	Male	1	176	141	141	119	137	70	174	113	56	27	40	38
1818		Arikara (0.4 - 0.1 ky)	4	Adult	Male	1	174	131	135	113	133	66	171	106	51	27	40	33
1819		Arikara (0.4 - 0.1 ky)	4	Adult	Male	1	189	134	139	109	138	67	187	112	52	30	40	29
1820		Arikara (0.4 - 0.1 ky)	4	Adult	Male	1	177	144	137	113	136	73	175	111	55	27	40	36
1821		Arikara (0.4 - 0.1 ky)	4	Adult	Male	1	182	142	129	119	143	73	179	108	54	30	42	37
1822		Arikara (0.4 - 0.1 ky)	4	Adult	Male	1	186	147	138	125	146	79	184	113	58	27	41	35
1823		Arikara (0.4 - 0.1 ky)	4	Adult	Male	1	173	138	127	111	126	63	171	106	47	27	41	35
1824		Arikara (0.4 - 0.1 ky)	4	Adult	Male	1	175	137	131	113	135	76	175	107	55	28	40	36
1825		Arikara (0.4 - 0.1 ky)	4	Adult	Male	1	190	150	141	123	151	69	190	111	52	28	42	33
1826		Arikara (0.4 - 0.1 ky)	4	Adult	Male	1	175	142	131	117	144	67	174	112	52	26	39	35
1827		Arikara (0.4 - 0.1 ky)	4	Adult	Male	1	188	140	134	118	144	75	187	111	55	29	43	36
1828		Arikara (0.4 - 0.1 ky)	4	Adult	Male	1	175	148	132	120	146	76	174	116	58	27	43	36
1829		Arikara (0.4 - 0.1 ky)	4	Adult	Male	1	185	141	137	118	146	75	184	117	59	25	42	37
1830		Arikara (0.4 - 0.1 ky)	4	Adult	Male	1	178	138	127	114	135	71	174	108	53	25	41	37
1831		Arikara (0.4 - 0.1 ky)	4	Adult	Male	1	175	140	122	111	138	70	173	102	55	29	39	32
1832		Arikara (0.4 - 0.1 ky)	4	Adult	Male	1	173	128	129	103	132	71	171	100	55	24	40	36
1833		Arikara (0.4 - 0.1 ky)	4	Adult	Male	1	190	142	137	117	146	75	186	112	55	27	39	33
1834		Arikara (0.4 - 0.1 ky)	4	Adult	Male	1	187	145	138	117	145	74	184	112	55	24	41	32
1835		Arikara (0.4 - 0.1 ky)	4	Adult	Male	1	175	140	133	113	136	69	174	110	50	25	39	34
1836		Arikara (0.4 - 0.1 ky)	4	Adult	Male	1	178	144	133	120	138	67	176	116	53	27	41	37
1837		Arikara (0.4 - 0.1 ky)	4	Adult	Male	1	181	147	128	119	146	71	178	102	54	27	41	35

SpecNo	OldNo	Location	PopCode	Age	Sex	SexCode	GOL	XCB	BBH	XFB	ZYB	NPH	NOL	FRC	NLH	NLB	OBB	OBH
1838		Arikara (0.4 - 0.1 ky)	4	Adult	Female	2	173	135	118	112	132	62	172	100	48	27	40	32
1839		Arikara (0.4 - 0.1 ky)	4	Adult	Female	2	163	135	123	109	129	72	163	103	52	25	38	35
1840		Arikara (0.4 - 0.1 ky)	4	Adult	Female	2	162	134	133	115	129	65	162	104	49	26	39	36
1841		Arikara (0.4 - 0.1 ky)	4	Adult	Female	2	171	137	131	108	128	64	168	106	48	27	37	34
1842		Arikara (0.4 - 0.1 ky)	4	Adult	Female	2	172	142	128	117	124	65	171	108	52	25	39	35
1843		Arikara (0.4 - 0.1 ky)	4	Adult	Female	2	166	135	121	107	133	64	166	99	50	24	39	33
1844		Arikara (0.4 - 0.1 ky)	4	Adult	Female	2	169	136	129	113	132	68	168	97	51	26	39	33
1845		Arikara (0.4 - 0.1 ky)	4	Adult	Female	2	174	139	130	115	143	66	168	100	52	27	40	37
1846		Arikara (0.4 - 0.1 ky)	4	Adult	Female	2	173	135	125	115	131	65	173	107	51	27	37	36
1847		Arikara (0.4 - 0.1 ky)	4	Adult	Female	2	170	137	126	115	130	69	170	109	49	25	38	34
1848		Arikara (0.4 - 0.1 ky)	4	Adult	Female	2	172	140	133	116	128	68	172	115	53	26	38	34
1849		Arikara (0.4 - 0.1 ky)	4	Adult	Female	2	171	134	131	111	130	69	170	111	51	25	40	33
1850		Arikara (0.4 - 0.1 ky)	4	Adult	Female	2	178	139	127	113	128	66	177	109	51	24	39	34
1851		Arikara (0.4 - 0.1 ky)	4	Adult	Female	2	168	138	137	117	134	67	165	108	50	29	38	36
1852		Arikara (0.4 - 0.1 ky)	4	Adult	Female	2	181	142	123	118	126	76	182	117	55	26	41	36
1853		Arikara (0.4 - 0.1 ky)	4	Adult	Female	2	175	141	125	119	138	67	174	111	52	30	40	37
1854		Arikara (0.4 - 0.1 ky)	4	Adult	Female	2	176	142	128	115	135	72	175	106	51	24	40	35
1855		Arikara (0.4 - 0.1 ky)	4	Adult	Female	2	162	133	124	105	127	69	161	100	49	25	40	36
1856		Arikara (0.4 - 0.1 ky)	4	Adult	Female	2	186	136	125	112	133	72	186	107	51	26	42	37
1857		Arikara (0.4 - 0.1 ky)	4	Adult	Female	2	179	131	123	112	133	74	178	103	54	26	40	36
1858		Arikara (0.4 - 0.1 ky)	4	Adult	Female	2	178	147	125	115	135	65	177	108	48	24	41	38
1859		Arikara (0.4 - 0.1 ky)	4	Adult	Female	2	167	128	120	108	122	62	167	98	47	25	37	33
1860		Arikara (0.4 - 0.1 ky)	4	Adult	Female	2	170	132	133	109	130	70	170	103	51	27	40	32
1861		Arikara (0.4 - 0.1 ky)	4	Adult	Female	2	163	126	135	105	126	71	163	102	50	23	38	33
1862		Arikara (0.4 - 0.1 ky)	4	Adult	Female	2	165	128	124	110	127	60	164	104	46	24	39	33
1863		Arikara (0.4 - 0.1 ky)	4	Adult	Female	2	166	140	124	122	130	70	166	109	50	26	41	35
1864		Arikara (0.4 - 0.1 ky)	4	Adult	Female	2	170	143	123	113	135	68	168	108	53	28	39	32
3065		Santa Cruz (0.4 ky)	5	Adult	Male	1	179	138	124	113	139	78	178	104	54	26	42	37
3066		Santa Cruz (0.4 ky)	5	Adult	Male	1	176	140	122	111	135	63	173	102	47	22	41	35
3067		Santa Cruz (0.4 ky)	5	Adult	Male	1	171	130	128	110	135	68	169	104	50	26	39	36
3068		Santa Cruz (0.4 ky)	5	Adult	Male	1	182	142	137	113	134	73	178	120	51	22	38	33
3069		Santa Cruz (0.4 ky)	5	Adult	Male	1	176	134	127	111	129	68	173	108	48	23	39	35
3070		Santa Cruz (0.4 ky)	5	Adult	Male	1	174	136	122	104	128	64	173	109	47	23	38	35
3071		Santa Cruz (0.4 ky)	5	Adult	Male	1	183	131	129	109	140	68	179	104	52	27	42	37
3072		Santa Cruz (0.4 ky)	5	Adult	Male	1	181	141	129	113	138	72	177	108	52	25	39	34
3073		Santa Cruz (0.4 ky)	5	Adult	Male	1	182	146	129	116	137	74	180	115	55	25	40	38
3074		Santa Cruz (0.4 ky)	5	Adult	Male	1	183	143	131	118	140	72	181	115	52	26	43	33
3075		Santa Cruz (0.4 ky)	5	Adult	Male	1	177	135	130	115	138	70	173	109	55	24	42	39

SpecNo	OldNo	Location	PopCode	Age	Sex	SexCode	GOL	XCB	BBH	XFB	ZYB	NPH	NOL	FRC	NLH	NLB	OBB	OBH
3076		Santa Cruz (0.4 ky)	5	Adult	Male	1	181	136	127	109	135	65	177	105	49	24	40	36
3077		Santa Cruz (0.4 ky)	5	Adult	Male	1	177	150	133	122	143	64	174	110	49	26	37	33
3078		Santa Cruz (0.4 ky)	5	Adult	Male	1	183	133	127	115	138	68	181	108	52	23	40	37
3079		Santa Cruz (0.4 ky)	5	Adult	Male	1	186	140	129	120	135	67	181	111	51	26	40	36
3080		Santa Cruz (0.4 ky)	5	Adult	Male	1	189	142	129	112	145	72	187	115	55	28	41	36
3081		Santa Cruz (0.4 ky)	5	Adult	Male	1	181	137	122	106	133	67	180	105	49	23	39	32
3082		Santa Cruz (0.4 ky)	5	Adult	Male	1	180	142	125	119	138	70	177	108	53	26	40	37
3083		Santa Cruz (0.4 ky)	5	Adult	Male	1	183	136	117	107	128	66	181	104	46	27	39	34
3084		Santa Cruz (0.4 ky)	5	Adult	Male	1	181	144	129	114	141	71	177	114	52	24	42	35
3085		Santa Cruz (0.4 ky)	5	Adult	Male	1	182	132	123	109	128	65	179	111	50	26	40	36
3086		Santa Cruz (0.4 ky)	5	Adult	Male	1	176	140	131	112	132	68	172	110	51	24	40	34
3087		Santa Cruz (0.4 ky)	5	Adult	Male	1	176	132	127	107	129	60	175	106	48	24	40	36
3088		Santa Cruz (0.4 ky)	5	Adult	Male	1	182	139	125	114	136	69	180	106	49	26	41	34
3089		Santa Cruz (0.4 ky)	5	Adult	Male	1	180	144	131	118	132	61	175	119	49	26	38	34
3090		Santa Cruz (0.4 ky)	5	Adult	Male	1	184	145	141	125	146	69	179	113	52	25	42	36
3091		Santa Cruz (0.4 ky)	5	Adult	Male	1	175	133	123	109	129	64	173	105	45	24	39	33
3092		Santa Cruz (0.4 ky)	5	Adult	Male	1	179	144	130	117	141	71	177	111	52	25	39	34
3093		Santa Cruz (0.4 ky)	5	Adult	Male	1	182	151	134	119	142	71	178	117	50	28	44	36
3094		Santa Cruz (0.4 ky)	5	Adult	Male	1	177	138	134	113	137	71	173	108	52	25	40	35
3095		Santa Cruz (0.4 ky)	5	Adult	Male	1	175	137	123	112	136	65	172	108	48	27	39	30
3096		Santa Cruz (0.4 ky)	5	Adult	Male	1	183	140	133	113	142	72	178	111	53	26	40	36
3097		Santa Cruz (0.4 ky)	5	Adult	Male	1	180	142	128	114	140	63	177	108	47	25	39	33
3098		Santa Cruz (0.4 ky)	5	Adult	Male	1	174	133	130	110	128	64	173	107	47	24	40	32
3099		Santa Cruz (0.4 ky)	5	Adult	Male	1	192	139	127	109	136	73	188	112	51	23	42	35
3100		Santa Cruz (0.4 ky)	5	Adult	Male	1	176	143	139	116	139	68	174	108	48	22	40	34
3101		Santa Cruz (0.4 ky)	5	Adult	Male	1	178	145	138	121	149	72	176	116	51	22	41	36
3103		Santa Cruz (0.4 ky)	5	Adult	Male	1	179	150	125	122	139	68	176	110	50	22	40	36
3104		Santa Cruz (0.4 ky)	5	Adult	Male	1	181	138	135	112	137	73	180	113	54	23	40	36
3105		Santa Cruz (0.4 ky)	5	Adult	Male	1	182	151	135	120	147	72	179	116	53	27	42	37
3106		Santa Cruz (0.4 ky)	5	Adult	Male	1	178	138	129	112	138	72	177	109	52	24	42	34
3107		Santa Cruz (0.4 ky)	5	Adult	Male	1	176	140	133	115	139	72	174	113	52	22	42	38
3108		Santa Cruz (0.4 ky)	5	Adult	Male	1	191	138	130	117	139	66	187	118	50	26	41	37
3109		Santa Cruz (0.4 ky)	5	Adult	Male	1	170	134	127	107	131	64	167	103	48	24	39	33
3110		Santa Cruz (0.4 ky)	5	Adult	Male	1	181	139	125	111	134	69	178	114	49	26	41	32
3111		Santa Cruz (0.4 ky)	5	Adult	Male	1	176	140	127	113	133	74	174	110	55	24	38	36
3112		Santa Cruz (0.4 ky)	5	Adult	Male	1	185	143	133	123	143	73	182	110	53	25	41	36
3113		Santa Cruz (0.4 ky)	5	Adult	Male	1	175	143	128	113	133	68	174	104	49	24	40	38
3114		Santa Cruz (0.4 ky)	5	Adult	Male	1	185	144	133	114	142	72	181	108	54	28	40	35

SpecNo	OldNo	Location	PopCode	Age	Sex	SexCode	GOL	XCB	BBH	XFB	ZYB	NPH	NOL	FRC	NLH	NLB	OBB	OBH
3115		Santa Cruz (0.4 ky)	5	Adult	Male	1	179	143	131	120	141	77	177	109	54	26	41	38
3116		Santa Cruz (0.4 ky)	5	Adult	Male	1	177	141	126	111	137	69	175	108	51	26	41	36
3117		Santa Cruz (0.4 ky)	5	Adult	Female	2	170	137	120	107	123	63	168	103	47	23	36	32
3118		Santa Cruz (0.4 ky)	5	Adult	Female	2	176	139	134	107	131	68	175	106	51	22	42	35
3119		Santa Cruz (0.4 ky)	5	Adult	Female	2	160	132	122	104	123	63	159	103	44	22	40	33
3120		Santa Cruz (0.4 ky)	5	Adult	Female	2	169	132	123	105	123	66	167	101	46	21	39	33
3121		Santa Cruz (0.4 ky)	5	Adult	Female	2	167	132	125	109	128	70	165	103	48	24	39	36
3122		Santa Cruz (0.4 ky)	5	Adult	Female	2	178	142	129	109	129	65	177	109	47	24	38	35
3123		Santa Cruz (0.4 ky)	5	Adult	Female	2	177	135	124	113	122	59	173	107	41	25	37	31
3124		Santa Cruz (0.4 ky)	5	Adult	Female	2	171	141	122	110	128	68	171	102	49	22	40	36
3125		Santa Cruz (0.4 ky)	5	Adult	Female	2	170	130	121	111	120	64	169	99	45	23	37	34
3126		Santa Cruz (0.4 ky)	5	Adult	Female	2	163	130	116	108	120	57	159	96	43	22	38	34
3127		Santa Cruz (0.4 ky)	5	Adult	Female	2	168	124	117	99	120	63	165	97	46	22	38	34
3128		Santa Cruz (0.4 ky)	5	Adult	Female	2	174	135	121	106	128	61	173	104	46	22	38	35
3129		Santa Cruz (0.4 ky)	5	Adult	Female	2	180	139	120	105	125	64	179	105	45	24	38	35
3130		Santa Cruz (0.4 ky)	5	Adult	Female	2	184	132	126	104	128	69	180	109	50	22	36	34
3131		Santa Cruz (0.4 ky)	5	Adult	Female	2	180	144	128	114	127	66	177	113	48	22	39	36
3132		Santa Cruz (0.4 ky)	5	Adult	Female	2	168	132	121	107	121	60	168	105	44	24	36	32
3133		Santa Cruz (0.4 ky)	5	Adult	Female	2	181	136	129	109	121	67	181	105	48	25	37	35
3134		Santa Cruz (0.4 ky)	5	Adult	Female	2	172	133	123	112	133	66	170	104	48	23	39	33
3135		Santa Cruz (0.4 ky)	5	Adult	Female	2	175	132	125	111	125	65	175	109	47	23	39	33
3136		Santa Cruz (0.4 ky)	5	Adult	Female	2	175	134	124	109	131	66	174	105	48	22	40	35
3137		Santa Cruz (0.4 ky)	5	Adult	Female	2	172	136	123	110	126	69	171	106	47	24	38	33
3138		Santa Cruz (0.4 ky)	5	Adult	Female	2	166	129	123	105	125	62	165	103	42	24	37	34
3139		Santa Cruz (0.4 ky)	5	Adult	Female	2	174	138	126	110	130	70	174	105	48	24	42	36
3140		Santa Cruz (0.4 ky)	5	Adult	Female	2	171	140	125	109	131	67	171	106	48	21	38	35
3141		Santa Cruz (0.4 ky)	5	Adult	Female	2	176	137	125	115	126	62	174	107	45	26	37	34
3142		Santa Cruz (0.4 ky)	5	Adult	Female	2	173	139	123	110	133	65	173	108	48	24	39	37
3143		Santa Cruz (0.4 ky)	5	Adult	Female	2	173	141	118	111	128	63	173	101	49	24	39	35
3144		Santa Cruz (0.4 ky)	5	Adult	Female	2	167	139	124	111	129	63	164	104	46	22	37	36
3145		Santa Cruz (0.4 ky)	5	Adult	Female	2	172	130	125	107	128	66	171	104	48	24	40	35
3146		Santa Cruz (0.4 ky)	5	Adult	Female	2	166	132	127	109	127	65	165	102	48	24	39	34
3147		Santa Cruz (0.4 ky)	5	Adult	Female	2	178	135	125	109	128	62	175	109	44	25	37	35
3148		Santa Cruz (0.4 ky)	5	Adult	Female	2	178	138	122	108	120	58	176	106	43	22	36	30
3149		Santa Cruz (0.4 ky)	5	Adult	Female	2	168	133	124	115	126	66	168	108	50	23	37	34
3150		Santa Cruz (0.4 ky)	5	Adult	Female	2	173	131	130	112	132	61	171	108	46	20	37	33
3151		Santa Cruz (0.4 ky)	5	Adult	Female	2	173	138	124	115	129	64	172	105	47	24	41	36
3152		Santa Cruz (0.4 ky)	5	Adult	Female	2	174	146	124	113	132	66	173	108	47	24	37	35

SpecNo	OldNo	Location	PopCode	Age	Sex	SexCode	GOL	XCB	BBH	XFB	ZYB	NPH	NOL	FRC	NLH	NLB	OBB	OBH
3153		Santa Cruz (0.4 ky)	5	Adult	Female	2	169	133	125	106	126	69	169	104	48	24	39	33
3154		Santa Cruz (0.4 ky)	5	Adult	Female	2	165	133	126	109	126	67	164	104	48	25	38	33
3155		Santa Cruz (0.4 ky)	5	Adult	Female	2	177	135	122	115	130	69	175	105	49	25	38	34
3156		Santa Cruz (0.4 ky)	5	Adult	Female	2	173	131	126	103	131	64	170	99	48	24	39	37
3157		Santa Cruz (0.4 ky)	5	Adult	Female	2	171	131	123	102	123	61	170	105	47	25	39	32
3158		Santa Cruz (0.4 ky)	5	Adult	Female	2	178	139	124	108	132	65	178	109	48	28	38	35
3159		Santa Cruz (0.4 ky)	5	Adult	Female	2	172	135	129	109	130	64	171	109	46	28	40	36
3160		Santa Cruz (0.4 ky)	5	Adult	Female	2	171	131	122	104	129	67	168	101	48	24	39	37
3161		Santa Cruz (0.4 ky)	5	Adult	Female	2	171	133	124	107	125	59	170	101	44	24	38	33
3162		Santa Cruz (0.4 ky)	5	Adult	Female	2	176	136	122	107	125	65	175	106	47	25	39	31
3163		Santa Cruz (0.4 ky)	5	Adult	Female	2	164	135	121	113	125	62	162	98	46	23	35	35
3164		Santa Cruz (0.4 ky)	5	Adult	Female	2	168	135	127	104	122	66	166	105	45	25	37	33
3165		Santa Cruz (0.4 ky)	5	Adult	Female	2	179	134	125	112	129	70	179	111	47	24	39	36
3166		Santa Cruz (0.4 ky)	5	Adult	Female	2	165	135	119	106	125	67	163	103	47	26	38	35
3167		Santa Cruz (0.4 ky)	5	Adult	Female	2	170	137	128	112	125	63	168	113	47	23	39	35
MN 629		Lagoa Santa (12 - 7 ky)	6		Male		182	134	131	118	137	67	178	109	50	23	40	33
MN 630		Lagoa Santa (12 - 7 ky)	6		Male		187	137	136	116	134	60	182	113	43	24	40	29
MN 804		Lagoa Santa (12 - 7 ky)	6		Male		187	132	128	114	138	71	185	108	53	26	41	36
MN 805		Lagoa Santa (12 - 7 ky)	6		Male		176	120	129	103	126	59	171	106	44	21	39	32
MN 807		Lagoa Santa (12 - 7 ky)	6		Male		182	132	137	110	133	61	178	104	48	26	39	33
MN 1355		Lagoa Santa (12 - 7 ky)	6		Male		185	127	132	105	131	67	183	111	48	27	39	33
MN 1357		Lagoa Santa (12 - 7 ky)	6		Male		184	124	138	106	128	63	180	112	49	24	40	32
HW 009		Lagoa Santa (12 - 7 ky)	6		Male		186	128	130	105	127	56	181	108	47	26	39	34
HW 010		Lagoa Santa (12 - 7 ky)	6		Male		183	125	139	108	133	72	180	110	56	26	39	30
HW AN14		Lagoa Santa (12 - 7 ky)	6		Male		183	126	135	107	133	60	181	114	43	25	39	33
CONFINS		Lagoa Santa (12 - 7 ky)	6		Male		179	126	131	112	129	76	178	110	54	31	36	37
SR1-1		Lagoa Santa (12 - 7 ky)	6		Male		196	125	134	108	134	72	190	114	55	27	45	35
SH-03		Lagoa Santa (12 - 7 ky)	6		Male		191	132	134	110	135	67	186	110	45	22	40	31
SH-04		Lagoa Santa (12 - 7 ky)	6		Male		182	128	137	114	140	70	179	111	50	24	40	33
SH-05		Lagoa Santa (12 - 7 ky)	6		Male		183	129	132	110	127	57	180	111	45	24	39	32
SH-09		Lagoa Santa (12 - 7 ky)	6		Male		182	129	139	114	134	65	180	112	47	25	41	35
SH-10		Lagoa Santa (12 - 7 ky)	6		Male		179	132	140	110	135	64	177	115	47	25	39	33
SH-16		Lagoa Santa (12 - 7 ky)	6		Male		180	131	139	113	134	61	177	110	49	25	40	33
MN 1353		Lagoa Santa (12 - 7 ky)	6		Female		181	134	127	111	129	67	178	108	49	26	39	33
MN 1388		Lagoa Santa (12 - 7 ky)	6		Female		174	130	131	108	128	60	170	108	44	25	38	34
MN 1959		Lagoa Santa (12 - 7 ky)	6		Female		185	126	133	108	123	61	181	109	46.5	26	39	33
HW S/N		Lagoa Santa (12 - 7 ky)	6		Female		176	133	136	115	130	60	174	110	46	25	37	32
HW 001		Lagoa Santa (12 - 7 ky)	6		Female		181	132.5	138	117	130	56	178	112	46	26.5	37.5	31

SpecNo	OldNo	Location	PopCode	Age	Sex	SexCode	GOL	XCB	BBH	XFB	ZYB	NPH	NOL	FRC	NLH	NLB	OBB	OBH
HW 004		Lagoa Santa (12 - 7 ky)	6		Female		179	129	136	105	127	61	175	109	45	24	38	33
HW 005		Lagoa Santa (12 - 7 ky)	6		Female		178	130	129	105	124	55	174	105	43	24	37	31
HW 006		Lagoa Santa (12 - 7 ky)	6		Female		183	124	138	108	132	59	180	102	49	24	36	30
SR1-6		Lagoa Santa (12 - 7 ky)	6		Female		171	118	131	104	120	54	169	106	42	20	34	30
SH-07		Lagoa Santa (12 - 7 ky)	6		Female		176	123	130	105	128	63	172	104	47	25	36	35
SH-08		Lagoa Santa (12 - 7 ky)	6		Female		185	131	136	116	133	66	180	113	50	27	39	34
CH10		Paleo Colombia (11 - 6 ky)	7		Male		187	122	130	107	134	72	186	108	52.5	28	42	33
TEQI02		Paleo Colombia (11 - 6 ky)	7		Male		174	129	133	103	125	74	173	109.5	51	22.5	36.5	31
TEQI03		Paleo Colombia (11 - 6 ky)	7		Male		192	130	133	109	131	67	186	112	48	25	37	33
TEQI12		Paleo Colombia (11 - 6 ky)	7		Male		192	126	139	106	140	68	187	112	50	27	42	31
TEQI13		Paleo Colombia (11 - 6 ky)	7		Male		194	134	137	108	139.5	69	188	114	51	23	38	36
TEQI16		Paleo Colombia (11 - 6 ky)	7		Male		180	136	135	112	138	63	179	106	48.5	26	36.5	33.5
CH07		Paleo Colombia (11 - 6 ky)	7		Female		174	131	125	109	130	61	171	107	44.5	22	37	33
CH13		Paleo Colombia (11 - 6 ky)	7		Female		184	128	132	109	119	65	184	107	46	27	41	35
GACHASN		Paleo Colombia (11 - 6 ky)	7		Female		188	125	131	105	109	67	186	104	51	26	38	36
TEQI07		Paleo Colombia (11 - 6 ky)	7		Female		186	133	136	110	134	67	185	111	54	25	38	35
TEQI18		Paleo Colombia (11 - 6 ky)	7		Female		172	132	130	107	130	63	170	106	47	21	39	31
TEQII02		Paleo Colombia (11 - 6 ky)	7		Female		173	131	128	109	123	64	172	107	46.5	23	38	34
TEQII03		Paleo Colombia (11 - 6 ky)	7		Female		183	127	130	109	127	75	182	109	52	23	39	38
TEQII01		Paleo Colombia (11 - 6 ky)	7		Female		179	134	128	105	127	64	177	107	51	26	38	34
SK 10030	SK 10030	Californian Coast (5 - 4 ky)	8		Male		186	140	131.5	105	131	72.56	134	112	51.1	25.69	38.55	31.84
WHMM #186	WHMM #186	Californian Coast (5 - 4 ky)	8		Male		183	140	131	103	134	63.7	132	110.5	48.91	23.65	38.48	33.79
WHMM #206	WHMM #206	Californian Coast (5 - 4 ky)	8		Male		193	150	129	112	135	70.89	142	111	54.27	26.22	44.44	37.99
SK 10015	SK 10015	Californian Coast (5 - 4 ky)	8		Male		182	139	140	106	136	75.84	140	112	54.82	22.87	40.8	36.36
WHMM #234	WHMM #234	Californian Coast (5 - 4 ky)	8		Male		194	141	134	114	148	68.68	142	116	55.56	26.14	40.58	37.65
WHMM #184	WHMM #184	Californian Coast (5 - 4 ky)	8		Female		180	134	128	101	133	61.07	132	113	47.21	20.57	36.59	35.46
WHMM #190	WHMM #190	Californian Coast (5 - 4 ky)	8		Male		185	141	133	110	144	73.45	140	113	50.43	20.78	25.32	33.13
SK 10037	SK 10037	Californian Coast (5 - 4 ky)	8		Male		195	146	144	113	140	71.49	140	115	51.65	24.77	39.93	33.51
SK 10103	SK 10103	Californian Coast (5 - 4 ky)	8		Female		182	141	131	101	133	65.91	134	108	48.84	26.75	37.52	33.25
SK 10104	SK 10104	Californian Coast (5 - 4 ky)	8		Male		186	133	135	101	140	66.4	140	105	50.54	25.27	35.92	34.16
S.G. No. 18	S.G. No. 18	Californian Coast (5 - 4 ky)	8		Female		188	141	130	104	131	77.51	132	113	52.08	21.66	37.8	30.83
SK 10019	SK 10019	Californian Coast (5 - 4 ky)	8		Male		191	139	145	106	133	67.06	142	116	49.71	22.07	38.75	33.6
SK 10041	SK 10041	Californian Coast (5 - 4 ky)	8		Male		188	141	144	110	140	68.2	142	112	52.59	24.65	40.33	35.5
SK 10043	SK 10043	Californian Coast (5 - 4 ky)	8		Male		179	140	134	106	141	65.47	132	112	54.1	23.43	38.14	36.7
SK 10116	SK 10116	Californian Coast (5 - 4 ky)	8		Male		182	138	132	100	136	72.28	141	106	51.92	23	36.19	31.89
SK 10017	SK 10017	Californian Coast (5 - 4 ky)	8		Male		180	137	138	101	134	67.89	136	110	50.46	23.29	36.97	37.02
WHMM #233	WHMM #233	Californian Coast (5 - 4 ky)	8		Female		176	140	120	102	129	66.4	139	108	47.33	24.17	34.05	31.91
WHMM #325	WHMM #325	Californian Coast (5 - 4 ky)	8		Male		182	139	140	101	140	71.71	140	113	51.15	22.06	35.35	33.06

SpecNo	OldNo	Location	PopCode	Age	Sex	SexCode	GOL	XCB	BBH	XFB	ZYB	NPH	NOL	FRC	NLH	NLB	OBB	OBH
MSQ1407	MSQ1407	Archaic Colombia (5 - 3 ky)	9		Male	1	190	131	142	110	133	70	185	117	50	25	38	35
MSQ1408	MSQ1408	Archaic Colombia (5 - 3 ky)	9		Male	1	184	134	145	112	133	68	181	113	53	27	36	38
MSQ1401	MSQ1401	Archaic Colombia (5 - 3 ky)	9		Female	2	172	131	138	107	124	60	171	109	46	25	36	33
CHIA05	CHIA05	Archaic Colombia (5 - 3 ky)	9		Female	2	184	122	134	103	130	71	182	106	52	28	37	33
AZ45823	AZ45823	Archaic Colombia (5 - 3 ky)	9		Male	1	190	130	130	109	132	77	187	107.5	52	27	36.5	32.5
AZ45833	AZ45833	Archaic Colombia (5 - 3 ky)	9		Male	1	179	145	143	129	147	69	176	105	50.5	26	37	32
AZ45842	AZ45842	Archaic Colombia (5 - 3 ky)	9		Male	1	183	127	134	103.5	123	71	181	113	52	27.5	37.5	32.5
AZ45848	AZ45848	Archaic Colombia (5 - 3 ky)	9		Male	1	184	131	137	114	132	71	181	111	51.5	26	36	34.5
AZ45861	AZ45861	Archaic Colombia (5 - 3 ky)	9		Male	1	191	125	141	104	135	72	186	112.5	56	26	39	35
AZ45822	AZ45822	Archaic Colombia (5 - 3 ky)	9		Female	2	178	131	131	106	119	61	176	108	44	26	36	32
AZ45840	AZ45840	Archaic Colombia (5 - 3 ky)	9		Female	2	185.5	126	135	105	115	70.5	183	109	52	24	38	35
AZ45841	AZ45841	Archaic Colombia (5 - 3 ky)	9		Female	2	179	124	129	108	120	58	176	107	46.5	24	35	32.5
AZ45849	AZ45849	Archaic Colombia (5 - 3 ky)	9		Female	2	185	132	138	110	129	63.5	182	113	46	27	37.5	34
AZ45851	AZ45851	Archaic Colombia (5 - 3 ky)	9		Female	2?	193	132	139	113	134	67	189	117	53	28	38	35
AZ45855	AZ45855	Archaic Colombia (5 - 3 ky)	9		Female	2	179	130.5	133	101	123	72	178	115.5	50.5	26.5	33.5	34.5
AZ45859	AZ45859	Archaic Colombia (5 - 3 ky)	9		Female	2?	197	130	138	115	136	63	194	122	51	27	39	34
AZ45863	AZ45863	Archaic Colombia (5 - 3 ky)	9		Female	2	173	128	126	103	117	62	171	107.5	46	22	36	32.5
AZ45869	AZ45869	Archaic Colombia (5 - 3 ky)	9		Female	2	180	120	132	102	125	62	178	106	49.5	26	39	35
AZ45872	AZ45872	Archaic Colombia (5 - 3 ky)	9		Female	2	183	125	127	106	119	57	180	113	44	27	38	31
AZ45874	AZ45874	Archaic Colombia (5 - 3 ky)	9		Female	2	181	129	128	109	112.5	56	177	110	39.5	21	35	31.5
ChanHol 3		Yucatan	10		Female	2	170	13	125	99	123	63	168	100	52	25	37	35
Las Palmas		Yucatan	10		Female	2	169	124	131	93	134	55	170.5	103	51	22	35	33
Muknal		Yucatan	10		Male	1	172	136	125	111	134	62	170.5	102	49	20	34	41
Maya		Yucatan	10		Male	1	174	134	120	93	134	65	173	103.5	51	22	32.5	39
