# Supplementary material for: New evidence for an early settlement of the Yucatán Peninsula, Mexico: The Chan Hol 3 woman and her meaning for the Peopling of the Americas
Source: PLoS One. 2020 Feb 5;15(2):e0227984. doi: 10.1371/journal.pone.0227984 (PMC7001910; doi:10.1371/journal.pone.0227984)
Supplement: S4 Table — For definition of the cranial variables see S3 Table. (PDF) [file pone.0227984.s006.pdf]

| PC Loadings | | |
| --- | --- | --- |
| Variable | PC1 | PC2 |
| GOL | 0.307574 | **-0.36476** |
| XCB | 0.223183 | **0.420818** |
| BBH | 0.314287 | **-0.36118** |
| XFB | 0.275161 | **0.430596** |
| ZYB | **0.396383** | 0.020524 |
| NPH | **0.352181** | -0.10807 |
| NOL | 0.050615 | **0.450502** |
| FRC | 0.320109 | -0.2105 |
| NLH | **0.375044** | -0.0202 |
| NLB | 0.24037 | -0.02636 |
| OBB | 0.24794 | 0.213619 |
| OBH | 0.187095 | 0.260587 |
