# Supplementary material for: New evidence for an early settlement of the Yucatán Peninsula, Mexico: The Chan Hol 3 woman and her meaning for the Peopling of the Americas
Source: PLoS One. 2020 Feb 5;15(2):e0227984. doi: 10.1371/journal.pone.0227984 (PMC7001910; doi:10.1371/journal.pone.0227984)
Supplement: S5 Table — (PDF) [file pone.0227984.s007.pdf]

**Specimen**	**Cranial Index**	**Superior Facial Index**	**Location**
Tecolote	67.6	41.5	Central Mexico
Peñon	70	47.8	Central Mexico
Chimalhuacan	71.1	53.1	Central Mexico
Balderas	73	48.5	Central Mexico
Asta_m	73.9	45	Central Mexico
Texcoco	74.9	50.7	Central Mexico
Texcal_f	75.5	46.4	Central Mexico
Astah_m	77.5	52.5	Central Mexico
Tehuacan_f	78.3	45.6	Central Mexico
Tepexpan	79	43	Central Mexico
Texcal_m	80	54.6	Central Mexico
Tlahuac	80.8	50	Central Mexico
Kennewick	74.1	56.3	N American, USA

Spirit Cave	70.3	50.7	N American, USA
Sumidero 1	69.1	49.6	S American, Lagoa Santa, Brazil
Sumidero 2	70.8	48.5	S American, Lagoa Santa, Brazil
Sumidero 3	72.8	45.5	S American, Lago Santa, Brazil
Sumidero 4	81.9	42.7	S American, Lagoa Santa Brazil
Palmas	76.2	44	Yucatan Peninsula
Hoyo Negro	76.9	47.4	Yucatan Peninsula
Muknal	78.3	45.3	Yucatan Peninsula
Chan Hol 3	76	51	Yucatan Peninsula
