# Supplementary material for: New evidence for an early settlement of the Yucatán Peninsula, Mexico: The Chan Hol 3 woman and her meaning for the Peopling of the Americas
Source: PLoS One. 2020 Feb 5;15(2):e0227984. doi: 10.1371/journal.pone.0227984 (PMC7001910; doi:10.1371/journal.pone.0227984)
Supplement: S6 Table — The data are normalized to standard SRM987. (PDF) [file pone.0227984.s008.pdf]

Lab-Code	Sample	Description	Date	$^{87}Sr/^{86}Sr$ (±2σ-mean)	Meas. Blocks#
PUA-Sr-9685	NBS987	Standard	22.11.2018	0.710265 ± 0.000014	9/10
PUA-Sr-9685	NBS987	Standard	22.11.2018	0.710254 ± 0.000018	5/10
PUA-Sr-9685	NBS987	Standard	22.02.2019	0.710269 ± 0.000032	8/10
PUA-Sr-9685	NBS987	Standard	07.03.2019	0.710292 ± 0.000026*	10/10
PUA-Sr-N987	NBS987	Standard	08.03.2019	0.710268 ± 0.00001	10/10
PUA-Sr-9610	Chanhol-3	3rd molar, left	22.02.2019	0.708878 ± 0.000042	7/10
	NBS987	Standard	Average	0.710264	(N=4)
	* outlier		2s-mean	0.000006	

* outlier

1 Block corresponds to 10 individual isotope ratio measurements (scans)
